# Integrated analysis of genome, metabolome, and transcriptome reveals a bHLH transcription factor potentially regulating the accumulation of flavonoids involved in carrot resistance to Alternaria leaf blight

**DOI:** 10.1371/journal.pone.0336995

**Published:** 2025-11-19

**Authors:** Claude Emmanuel Koutouan, Marie Louisa Ramaroson, Angelina El Ghaziri, Laurent Ogé, Abdelhamid Kebieche, Raymonde Baltenweck, Patricia Claudel, Philippe Hugueney, Anita Suel, Sébastien Huet, Linda Voisine, Mathilde Briard, Jean Jacques Helesbeux, Latifa Hamama, Valérie Le Clerc, Emmanuel Geoffriau

**Affiliations:** 1 Institut Agro, Université d’Angers, INRAE, IRHS, SFR QUASAV, Angers, France; 2 Université de Strasbourg, INRAE, SVQV UMR-A, Colmar, France; 3 Université d’Angers, Faculté de santé – Département Pharmacie, Angers, France; ICAR - National Rice Research Institute, INDIA

## Abstract

Resistance of carrot to Alternaria leaf blight (ALB) caused by *Alternaria dauci* is a complex and quantitative trait. Numerous QTL for resistance (rQTLs) to ALB have been identified but the underlying mechanisms remain largely unknown. Some rQTLs have been recently proposed to be linked to the flavonoid content of carrot leaves. In this study, we performed a metabolic QTL analysis and shed light on the potential mechanisms underlying the most significant rQTL, located on carrot chromosome 6 and accounting for a large proportion of the resistance variation. The flavonoids apigenin 7-*O*-rutinoside, chrysoeriol 7-*O*-rutinoside and luteolin 7-*O*-rutinoside were identified as strongly correlated with resistance. The combination of genetic, metabolomic and transcriptomic approaches led to the identification of a gene encoding a bHLH162-like transcription factor, which may be responsible for the accumulation of these rutinosylated flavonoids. Transgenic expression of this bHLH transcription factor led to an over-accumulation of flavonoids in carrot calli, together with significant increase in the antifungal properties of the corresponding calli extracts. Altogether, the bHLH162-like transcription factor identified in this work is a strong candidate for explaining the flavonoid-based resistance to ALB in carrot.

## Introduction

Carrot production is challenged by various pests and pathogens that can affect either the roots or the foliage [1,2]. Among these biotic stresses, Alternaria leaf blight (ALB), caused by the necrotrophic fungus *Alternaria dauci (A. dauci),* is considered the most damaging foliar disease worldwide [3–6]. This disease is characterized by brown lesions on the surface of leaves, which can lead to complete foliar blight and potentially total yield loss in cases of severe infection. As highlighted by Le Clerc and Briard [6], current ALB management relies mainly on chemical control, with applications partially optimized by predictive models. Despite extensive research, practices based on biological agents have not yet been translated into operationally viable solutions. Cultural practices and seed treatments provide limited and inconsistent efficacy. In order to achieve sustainable disease management, varietal resistance is a key element, which must be integrated with complementary methods. To date, no carrot genotype has been identified as completely resistant, and no major resistance gene has been identified. Our long-standing objective is to provide breeders with sources of high resistance and knowledge about resistance mechanisms, thereby filling a gap in the literature.

In previous studies, we identified highly resistant sources, but these resistances are polygenic, making introgression challenging [7]. Although resistance to ALB is governed by multiple loci, the heritability of disease resistance over three years was remarkably high, at approximately 71%, leading to highly consistent detection of rQTL mapped in the carrot genome [8–11]. Our current goal is to decipher the underlying mechanisms in order to identify genes and metabolites that could be targeted by breeders.

Resistance QTLs may be associated with various mechanisms, including morphological or physiological traits, genes involved in basal defense, minor resistance genes, signal transduction, secondary metabolite synthesis, or currently undefined mechanisms [12]. Several studies have successfully explored the relationship between rQTLs and specific metabolites in resistance to fungal pathogens. For example, co-localization between rQTLs and mQTLs in *Arabidopsis* suggested that camalexin was involved in resistance against *Botrytis cinerea* [13]. Hamzehzhargani *et al*. [14] identified 22 resistance-related metabolites associated with a rQTL on chromosome 2DL of wheat, conferring resistance to Fusarium head blight (FHB), using recombinant inbred lines (RILs) carrying alleles with opposing effects for the QTL. Gunnaiah *et al*. [15] found that near isogenic lines (NILs) carrying the resistant *Fhb1* QTL allele accumulated higher levels of hydroxycinnamic acid amides, phenolic glucosides, and flavonoids compared to the susceptible ones. More recently, in carrot, we employed a co-localization approach between rQTLs and mQTLs to identify fungitoxic terpenes and associated candidate genes underlying resistance QTLs mainly located on chromosomes 4 and 8 [9].

By comparing several carrot genotypes with varying levels of resistance to ALB, Koutouan *et al*. [16] found that the resistant genotypes accumulated higher levels of a specific set of metabolites compared to the highly susceptible genotype H1. Among these metabolites, they identified not only the terpenes that were later related to rQTLs, as described above, but also twelve flavonoids as discriminant compounds. The resistant genotypes I2 and Bolero were characterized as rich in flavonoids compared to H1.

In the literature, as reviewed by Ramaroson *et al*. [17] flavonoids represent a large and structurally diverse family of low-molecular-weight secondary metabolites synthesized by plants through specialized metabolic pathways. Alongside phenylpropanoids, they are classified within the broader group of polyphenolic compounds, all sharing a canonical C6-C3-C6 carbon skeleton. Their biosynthetic pathway is initiated by the conversion of naringenin chalcone into the flavanone naringenin, which constitutes a central precursor for multiple flavonoid subfamilies, including flavones, flavanols, anthocyanidins, and isoflavonoids. Subsequent structural diversification is achieved through extensive modifications of the flavonoid backbone, such as C- or O-methylation, sulfation, or glycosylation, thereby generating a vast array of derivatives. For instance, the flavone apigenin may undergo hydroxylation at the 3′ position of the B-ring to yield luteolin, which can subsequently be methylated to form chrysoeriol. Owing to this remarkable structural plasticity, flavonoids fulfill a wide spectrum of physiological and ecological roles in plants, notably in mediating responses to biotic stresses and interactions with the microbiome [17–23]. They are described as acting indirectly as signaling molecules, or directly through the toxic effect of phytoanticipins and phytoalexins. For instance, they were linked to the constitutive resistance of barley or wheat to FHB [24,25] or found to inhibit the fungal growth of *Aspergillus niger* and *Cladosporium cucumerinum* [26]. Very recently, based on a strong correlation between metabolite content and resistance level, three of these flavonoids were proposed by Ramaroson *et al*. [27] as potential biomarkers of resistance to ALB. However, no functional evidence has yet been provided to confirm their role. The present study therefore aims to test the hypothesis that, like terpenes, flavonoids contribute to carrot resistance to *A. dauci* by characterizing the mechanism underlying a major rQTL located on chromosome 6 (chr6), consistently detected across years, called RL14 in [9] and accounting for 20% of resistance variation. This strategy aims to enable the pyramiding of complementary mechanisms within elite cultivars, thereby achieving stronger and more durable resistance.

For this purpose, flavonoids previously identified by Koutouan *et al*. [16] were quantified in a segregating population following *A. dauci* infection under field conditions. Co-localization between mQTLs and rQTLs was investigated. Transcriptomic analyses were conducted to identify differentially expressed genes (DEG) between the two parental genotypes – one resistant and one susceptible – of the segregating population. The DEG list was then compared to the genome annotation within the mQTL-rQTL co-localization region. Transformation using *Agrobacterium tumefaciens* was carried out to investigate the role of identified candidate genes in candidate flavonoid accumulation and in carrot resistance to ALB.

## Materials and methods

### Plant materials

Two accessions – H1 (susceptible to *A. dauci*) and I2 (partially resistant) – both being parental lines of a segregating population named PC2, two commercial hybrids – Presto (susceptible to *A. dauci*) and Bolero (partially resistant) – and 120 F2:3 progenies of PC2 population were used in the present study [8,9,11,16]. Their level of resistance was evaluated by Koutouan *et al*. [9].

### Experimental designs and crop conditions

For mQTL detection, two field trials were conducted in 2015 at Blagon (Latitude 44.7835; Longitude −0.9319, Gironde, France) and in 2016 at Ychoux (Latitude 44.3333; longitude −0.9667 Les Landes, France). Each PC2 progeny was sown in two replicates of 180 seeds, while each parental line and Bolero were sown in ten replicates. In order to homogenize the disease pressure, Presto was sown along the entire length of the seedbed central rows. Two blocks were established on each side of this Presto row, with all replicates of progenies and parental lines randomly distributed within them. As described by Koutouan *et al*. [16], both trials were performed under farmer conditions in carrot production areas where *A. dauci* pressure is very high.

In order to harvest samples under optimal conditions for RNA extractions and microarray analysis, another trial including the two parental lines (H1 and I2) was conducted in 2017, close to our laboratory, under tunnel conditions at Angers (Latitude 47.4711; Longitude −0.5518 Maine et Loire, France). Four replicates of each line with 360 seeds were sown. The entire trial was bordered by Presto and inoculated with P2 *A. dauci* strain as described in [28,29].

### Metabolomic sampling design

For each trial, eight plants per replicate were harvested a few days after natural infestation or inoculation. The following steps were as described by Koutouan *et al*. [16]. Briefly, the plants were transported in cold conditions to the laboratory where two intermediate leaves of the eight plants of a sample were bulked, ground in a mortar with liquid nitrogen to obtain fresh powder then stored at −80°C. About 1 g of each fresh powder was freeze-dried and ground again with iron beads using a MM2 Retsch mixer-mill to obtain a fine powder.

### Targeted analysis of flavonoids

Analysis of flavonoids were performed by UHPLC-ESI-MS as described by Koutouan *et al*. [16]. Briefly, 10 mg of fine freeze-dried leaf powder from each replicate were extracted with methanol containing phenyl glucoside as internal standard. For the analysis of flavonoids in calli, approximately 1 g of callus tissue was placed in a 2 mL screw-cap tube and freeze-dried. A 50 mg sample of the resulting dry material was then extracted exactly as previously described.

Flavonoids identified in parental lines by Koutouan *et al*. [16] were quantified using the Xcalibur software. The exact *m/z* and retention time of each selected metabolite were used and the integration of each peak was checked manually before validation. The peak areas were considered as raw data. Using RStudio 1.0.136 [30], a “check-up phase” consisting in reviewing the homogeneity of replicates, residual normality and variance homogeneity was performed from the raw data. For each metabolite, to assess the reproducibility of the raw values obtained from biological replicates in the field, an R script was developed to identify the number of genotypes presenting a ratio of raw values above 2 or 3. Except for heritability calculations, genotypes with ratios higher than 3 for most of the metabolites were discarded from further analyses, or the values were changed into missing data when only a few metabolites were concerned.

Broad sense heritability (*H*^*2*^) was estimated as H^*2*^* =* *σG*^*2*^*/* σ*P*^*2*^, where *σG*^*2*^ is the genotypic variance and *σP*^*2*^ the phenotypic one. The phenotypic variance was calculated as *σP*^*2*^* =* *σG² + σGY²/Y + σε²/rY* where *σGY²* is the genotype:environment variance, *Y* is the number of year, *σε²* is the residual variance and *r* the number of replicates.

For correlation and QTL analyses, flavonoid data were the means of autoscaled data (2015 and 2016), i.e., centered to mean and scaled to the standard deviation of the metabolite in each genotype [31].

### Correlation between metabolite accumulation and disease score

Spearman correlation was calculated between flavonoid contents obtained here and disease scores released by Koutouan *et al*. [9] on the PC2 progeny plants (same fields, same plants). Significance of each correlation was estimated with p. value computed by student t. test using RStudio 1.0.136 [30].

### Metabolite QTL (mQTL) detection

These analyses were performed as described by Le Clerc *et al*. [11] and Koutouan *et al.* [9] by regression interval mapping using MCQTL-5.2.6-Linux.sh software [32]. A threshold value for QTL detection and cofactors selection were computed under an *F* test with 1000 permutations. Marker cofactors were selected in a forward method with 90% of the detection threshold value. Then, QTLs were detected with the iterative QTL mapping procedure according to the detection threshold value. A QTL was detected when the LOD (logarithm of odds) exceeded the threshold. A 1 and 1.5 LOD support interval suitable for 95% confidence interval were computed for all QTLs. The phenotypic variation explained by each QTL separately and by all QTLs were calculated and referred as R^2^ and global R^2^, respectively.

### *In silico* analysis of genes located under co-localization area

SSR molecular markers published by Le Clerc *et al*. [11] flanking mQTL-rQTL co-localization regions were aligned to the carrot genome sequence [33] using the Geneious 10.2.3 software (https://www.geneious.com, [34]). The locus number of each gene inside the co-localization area was extracted.

### Transcriptomic analysis

Fresh ground leaves of each sample of the two H1 and I2 lines were ground in a mortar with liquid nitrogen to obtain very fine powder. RNA was extracted following the protocol of the NucleoSpin® RNA Plus kit (Macherey-Nagel 2016, REF 740984 [35]), quantified using a NanoDrop ND-1000 (NanoDrop Technologies), and RNA quality was assessed with an Agilent 2100 Bioanalyzer. After extraction, 100 ng of RNA from each replicate were amplified and labeled using Agilent kit (ref: 5190−2306) and following the corresponding procedure [36]. Then, the cRNA was purified with Rneasy Mini Kit (Qiagen, ref: 74106) and then hybridized onto the Agilent- *Daucus_carota* v1 chip described by Koutouan *et al*. [9] (Agilent ref: G4862A). After hybridization, washing steps were performed and the blade was scanned using the InnoScan 710 (Innopsys) scanner. For the comparison of H1 and I2 samples, two biological replicates with two technical repetitions per replicate were analyzed in dye-swap as described by Gagnot *et al.* [37]. Data extraction and analyses were exactly similar to those previously described by Koutouan *et al* [9].

### Functional validation: Transformation and characterization of transformants

#### Induction of callogenesis and somatic embryogenesis.

Callogenesis and somatic embryogenesis were performed from seedlings according to our previous work [38,39], with slight modifications. Seeds of H1 and I2 were surface sterilized and germinated on moist filter paper. Seedlings were then transferred to B5 medium supplemented with 30 g/L sucrose and 3 g/L phytagel, and grown at 23/19 °C until leaf formation. Petiole fragments (4–5 mm) were cultured on the same B5 medium with 1 mg/L 2,4-D in the dark to induce callus. Calli were subcultured every 3 weeks on half-strength B5 with reduced 2,4-D (0.1 mg/L) to obtain friable callus. Embryogenic calli were then transferred to hormone-free half-strength B5 medium under low light for 6 weeks to induce somatic embryo formation. These friable embryogenic calli were used as the target material for *Agrobacterium tumefaciens*–mediated genetic transformation.

#### Gene cloning and transfection of *A. tumefaciens.*

The DNA sequence of the differentially expressed gene was synthesised and cloned into a pTwist entry vector by Twist Bioscience (San Francisco, California, USA). The entry vector was then introduced into *Escherichia coli* strain TOP10 by thermal shock at 42 °C for 30 seconds. *E. Coli* clones were selected after one day of culture on LB medium supplemented with kanamycin (50 µg/mL) at 37 °C. Plasmids were extracted using the Nucleospin plasmid extraction kit (Macherey-Nagel, Düren, Germany [35] and characterised by sequencing, using M13 universal primers (M13-F: 5’-GTAAAACGACGGCCAG-3’ and M13-R: 5’-CAGGAAACAGCTATGAC-3’). The gene of interest was then cloned into a pK7WG2D destination vector [40] using an LR Clonase II kit (Invitrogen, Waltham, Massachusetts, USA). Ligation products were transferred into *E.coli* strain TOP10 following the same procedure as described above and positive clones were selected on LB medium supplemented with spectinomycin (100 µg/mL) at 37 °C. Plasmids were extracted from the selected clones using the Nucleospin Plasmid Extraction Kit [35] and their sequence was confirmed by sequencing using the p35S primer (P35S_pK7WG2D_For 5’-AAGAAGACGTTCCAACCACG-3’). The destination vector was transferred into *Agrobacterium tumefaciens* strain EHA105 (pBBR1-MCS-5) by electroporation and selected after three days of culture on LB medium supplemented with rifampicin (10 µg/mL), gentamicin (10 µg/mL) and spectinomycin (100 µg/mL) at 28 °C.

#### Preparation of the bacterial suspensions and carrot cell transformation.

The preparation of bacterial solution was performed according to our previous work on rose [41] except slight modifications in antibiotics as followed: spectinomycin (100 µg/mL), gentamycin (10 µg/mL) and rifampicin (10 µg/mL).

Carrot embryogenic calli were exposed to *A. tumefaciens* suspension at OD_600_ = 0.1 and then co-cultured on Whatman paper placed on top of the culture medium in the dark at 19–23°C for 1 day. Transformant selection was subsequently initiated by transferring the calli on a fresh medium supplemented with 100 µg/mL kanamycin and 500 µg/mL cefotaxime, to discard non-transformed calli and *A. tumefaciens* respectively. Calli were sub-cultured every three weeks on a fresh B5 Gamborg ½ medium supplemented with 50 µg/mL kanamycin until callus masses homogeneously expressing GFP were observed by fluorescence microscopy.

#### Selection of transformation events for genotyping and phenotyping.

The fluorescence of the GFP in the calli was visualised using an SZX16 stereomicroscope (Olympus, Tokyo, Japan): λexcitation= [460–480nm], λemission=[495–540nm]. The 15 most homogeneously fluorescing callus masses were considered as 15 separate transformation events and spotted for multiplication. Calli not exposed to *A. tumefaciens* were used as a negative control (NT). Multiplication consisted of sub culturing of one callus mass in one Petri dish of B5 Gamborg ½ medium with 50 mg/L kanamycin. Multiplied events and NT calli were sub-cultured on a fresh medium 3 weeks before sampling. NT calli were cultured on a kanamycin-free medium to prevent their loss.

#### Gene expression analysis in carrot calli.

RNA extraction was carried out from 50 mg of fresh calli, using the NucleoSpin RNA Plus kit [35], according to the manufacturer’s protocol. Total extracted RNA was quantified using a NanoDrop ND-1000 spectrophotometer (NanoDrop Technologies, Wilmington, Delaware, USA). The absence of genomic DNA was verified by endpoint PCR (94 °C for 5 minutes, 35 cycles of (94 °C for 30s, 56 °C for 90s, 68 °C for 60s), 68 °C for 10 minutes, 4 °C for 5 minutes) using primer pairs targeting an intronic sequence of the *EF1-α* gene (EF1-α-int-F 5’- GCTTTACTTGCATTTACCCTTGGT-3’ and EF1-α-int-R 5’-ACCCAACCTTCTTCAAGTAAGATGA-3’). Insertion of the nptII gene was also verified by endpoint PCR.

Reverse transcription (RT) of RNA was performed on 2-µg total RNA in a 25-µL reaction volume using the M-MLV Reverse Transcriptase Kit (Promega, Madison, Wisconsin, USA). The reaction program was as follows: 42 °C for 60 minutes, 70 °C for 10 minutes and 4 °C for 10 minutes.

Quantitative real-time PCR (qRT-PCR) was performed on the obtained cDNA using the GoTaq®qPCR Master Mix kit (Promega, Madison, WIS, USA) according to the manufacturer’s protocol and using a Chromo4 Real-time PCR detector (Bio-Rad CFX connect Real-Time System, Inc., Hercules, CA, USA): 95 °C for 5 minutes, 40 times (95 °C for 3s, 60 °C for 1 minute), 60 °C to 95 °C for 5s and plate reading.

Three housekeeping genes *GAPDH* [0.2 µm], *α tubulin* [0.2 µm], and *actin* [0.1 µm] (primer sequences in Patent No. DI-RV-19–0075, an extension of the Patent No. WO/2011/161388) were used as reference genes to evaluate the expression level of the candidate gene [0.1 µm]. Three technical replicates per sample were performed.

Quantitative PCR data were retrieved from the Bio-Rad CFX Maestro1.1 software. Gene expression level was normalized with the expression of the housekeeping genes. The 2^ΔΔ^Cq method [42] was used to obtain the expression level of the target gene referring to its expression level in the empty vector modality of H1. For this purpose, the average expression level of 7 empty vector controls in H1 transformation events were calculated and used as a reference thereafter.

### Biological activity of callus extracts

#### Callus hydromethanolic extracts for biological activity assay.

According to availability, two or three samples of one modality were gathered to obtain enough material and weighed (1–2 g) then placed in a −80°C freezer until use. To obtain callus extracts a hydro-alcoholic solvent extraction (MeOH/H2O 75/25) was performed. For this, the calli were ground in an ice-cold mortar in the presence of a pinch of Fontainebleau sand. The crushed material was suspended in 10 mL of hydromethanolic solvent (MeOH/H_2_O 75/25 v/v), corresponding to the suspension volume of the powder and two rinses of the mortar. The solution obtained was placed in an ultrasonic bath for 20 minutes. The liquid extract was recovered and another 2 x 2.5 mL of residual extract from the rinsing of the material and the container was added. 10 µL of DMSO was then added to the suspension before the container was returned to the ultrasonic bath for another 20 minutes. The liquid extract was filtered through a 25-µm metal filter and completed with 3 x 2.5 mL of the wash. The extracts obtained were centrifuged twice at 8500 rpm for 5 minutes. The supernatant was collected and the solvent was removed under reduced pressure using a rotary evaporator with the heating bath at 40 °C. Dried callus extracts were resuspended in MeOH/H2O 53/1 v/v and filtered on cellulose (0.45 µM).

#### *In vitro* biological activity test of callus extracts against *A. dauci.*

Stock solutions of callus extracts were prepared at 100 mg/mL in Potato Dextrose Broth (PDB) with 1% DMSO. PDB with 1% DMSO was used as a control. Conidia were harvested from a 7-days sub-cultured *A. dauci* V8 medium as described by Pawelec *et al*. [28] by flooding sporulating mycelia with sterile distilled water and gently scrapping the surface of the medium. The suspension was filtered through sterile compress, to eliminate coarse mycelial fragments. The concentration of the conidial suspension was adjusted to 4 x 104 conidia/mL and Tween 20 was added to make up 0.05% of the suspension. A volume of 500 µL of the suspension was pipetted into small tubes and the conidia were sedimented using a benchtop centrifuge for 10 s. The supernatant was thereafter discarded and the pellet was resuspended in 500 µL of each of the corresponding compound solutions prepared above.

The solutions were gently homogenised using a vortex mixer, after which a 100 µL drop was placed on a microscope slide and covered with a coverslip. A total of 4 slides were prepared per modality as biological replications. Slides were then incubated in the dark at 22 °C for 5 hours and incubation was stopped by transfer to −80 °C.

Slides were observed with a microscope Leica DMR HC light microscope (ref: 1188054) coupled with a QImaging Retiga 2000R Colour CCD camera under x20 objective lens magnification.

Germination rate was estimated on 100 conidia randomly found at the central area of each slide. In this assessment, a conidium was considered germinated if it has at least one visible hypha regardless of its integrity. The hyphae were visually inspected to note their integrity. The conidia whose all hyphae were disrupted were also counted. These two evaluations were repeated on the four slides of each modality leading to a total of 3200 observations.

Statistics. All statistical analyses were performed in R v4.5.0, with a significance level set at 0.05. Whatever the analysis, ANOVA model assumptions were evaluated. When assumptions were verified (e.g., for Biological assay data or for Leaf metabolite data collected prior to transcriptomic analyses, that were log-transformed), a one-way ANOVA model was applied and Tukey’s HSD test was used for post hoc comparisons. When assumptions were not verified, even after transformation (e.g., for qPCR Data of gene expression or for Callus Metabolite data), data were analyzed with the Kruskal–Wallis test and Conover’s post hoc test, with p-values adjusted using the Benjamini–Hochberg method.

## Results

### Correlation of metabolite contents with resistance and mQTL-rQTL co-localization

Twelve flavonoids, previously described by Koutouan *et al*. [16], were targeted for this analysis, namely apigenin 7-O-glucoside (Api 7G), luteolin 7-O-glucoside (Lut 7G), chrysoeriol 7-O-glucoside (Chry 7G), apigenin 7-O-rutinoside (Api 7R), luteolin 7-O-rutinoside (Lut 7R), chrysoeriol 7-O-rutinoside (Chry 7R), apigenin 4’-O-glucoside (Api 4’G), luteolin 4’-O-glucoside (Lut 4’G), luteolin 7-O-glucuronide (Lut 7GR), apigenin 7-O-malonylglucoside (Api 7MG), luteolin 7-O-malonylglucoside (Lut 7MG), chrysoeriol 7-O-malonylglucoside (Chry 7MG).

**Fig 1:**
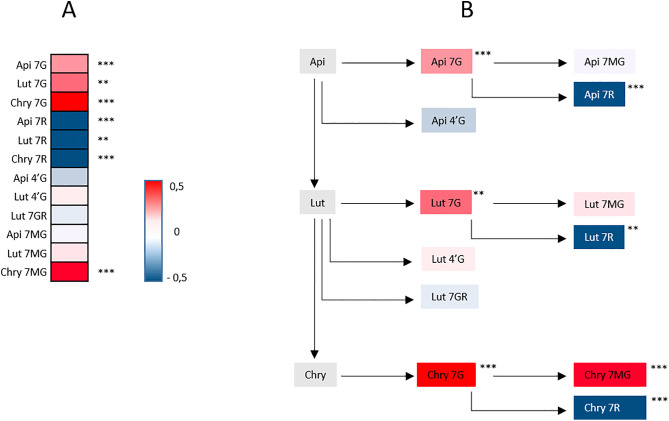
Studied flavonoid compounds. A (left) Correlation between disease severity and the accumulation level of twelve flavonoids. Flavonoid abbreviations are Api for Apigenin, Lut for Luteolin and Chry for Chrysoeriol; 7G for 7-*O* glucoside, 7R for 7-*O* rutinoside, 4’G for 4’-*O* glucoside, 7GR for 7-*O* glucuronide and 7MG for 7-*O* malonylglucoside. Red indicates a positive correlation and blue, a negative one. The intensity of colours indicates the level of correlation from −0.5 to 0.5. Spearman method in R v4.5.0 was used to calculate the correlation coefficient. Significance of correlation is given by p.value “*”0.01, “**”0.001, “***”0.0001. B (right) biosynthesis links between the twelve flavonoids as proposed by Koutouan *et al.* [16].

In order to eliminate fortuitous co-localizations between putative mQTLs and rQTLs, we first performed a correlation test between metabolite contents and disease scores. Seven metabolites were significantly correlated with the disease score. Among them, Api 7R, Lut 7R, and Chry 7R showed a strong negative correlation with disease severity, suggesting that higher accumulation of these compounds is associated with increased resistance. In contrast, the contents of Api 7G, Lut 7G, Chry 7G and Chry 7MG exhibited a significant positive correlation with disease score, suggesting a link to susceptibility (**Fig 1A**). All these flavonoids are derived from a common biosynthetic pathway, with apigenin as the main precursor (**Fig 1B**).

The compounds Api 4’G, Lut 4’G, Lut 7GR, Api 7MG and Lut 7MG, which were not correlated with disease score, were eliminated from subsequent analyses. The heritability for the accumulation of the seven correlated flavonoids was very high, ranging from 68% for Chry 7MG to 93% for Api 7R and Api 7G (**Table 1**).

**Table 1:** Heritability of seven flavonoid correlated with disease score.

Metabolite	H² = σG²/σP² (%)
Apigenin 7-O-glucoside (Api 7G)	93.1
Luteolin 7-O-glucoside (Lut 7G)	75.7
Chrysoeriol 7-O-glucoside (Chry 7G)	86.5
Apigenin 7-O-rutinoside (Api 7R)	92.7
Luteolin 7-O-rutinoside (Lut 7R)	89.0
Chrysoeriol 7-O-rutinoside (Chry 7R)	91.5
Chrysoeriol 7-O-malonylglucoside (Chry 7MG)	68.5

This very high heritability allowed an efficient mQTL detection. Indeed, as shown in **Table 2**, mQTLs for metabolite accumulation were detected for all seven metabolites. A total of 14 mQTLs were identified on chromosomes 1, 3, 4, and 6. The proportion of variance in metabolite accumulation explained by these mQTLs (global R²) was particularly high, ranging from approximately 40% to a maximum of 70%. Half of the mQTLs were located on chr6. The R² values of the mQTLs for Api 7R, Lut 7R and Chry 7R — the three metabolites negatively associated with disease severity, and thus positively associated with resistance — were particularly high in this genomic region (30.4%, 68.2% and 69.2%, respectively). The alleles associated with higher accumulation of these three metabolites originated from the resistant parent, I2, whereas the alleles linked to high flavonoid accumulation positively correlated with disease severity (Api 7G, Lut 7G, Chry 7G, Chry 7MG) were inherited from the susceptible parent, H1.

**Table 2:** List of mQTLs co-localized with rQTLs and exhibiting a significant correlation with disease score. mQTLs were detected over two years under field conditions in the PC2 segregating population. (SI = Support Interval; Chr = Chromosome; R² = Explained phenotypic variation; cM = centimorgan; H1 and I2 = carrot genotypes susceptible and resistant, respectively, to ALB). Bold numbers highlight details about QTLs located on chromosome 6.

Code of mQTL	Chr	1-LOD SI (cM)	Max position (cM)	R² (%)	Global R² (%)	Additive effect of allele	
						H1	I2
Lut 7R	**6**	**25.6–25.8**	**25.8**	**68.2**	68.2	−0.503	0.503
Lut 7G	1	12.1 - 49.2	47.3	8.7	37.1	0.144	−0.144
	4	0.9 - 50	34.7	9.1		0.136	−0.136
	**6**	**14.4 - 37.6**	**31.1**	**25.6**		0.249	−0.249
Api 7R	4	31.2 - 43.6	40.5	32	50	−0.321	0.321
	**6**	**25 - 28.2**	**25.8**	**30.4**		−0.301	0.301
Api 7G	4	34.6 - 47.7	40.5	40.7	52.7	−0.341	0.341
	**6**	**23.5 - 30.4**	**25.8**	**27.2**		0.245	−0.245
Chry 7R	1	40.1 - 66	41.3	12.6	71.1	0.131	−0.131
	**6**	**21.4–25.8**	**25.8**	**69.2**		−0.5023	0.5023
Chry 7G	1	37.2 - 50.4	41.3	28.6	52.5	0.263	−0.263
	**6**	**24.3 - 28.2**	**25.8**	**42.9**		0.328	−0.328
Chry 7MG	3	53.1 - 69.7	55.7	25.8	41.5	0.291	−0.291
	**6**	**11–26.1**	**25.8**	**25.1**		0.256	−0.256

**Fig 2:**
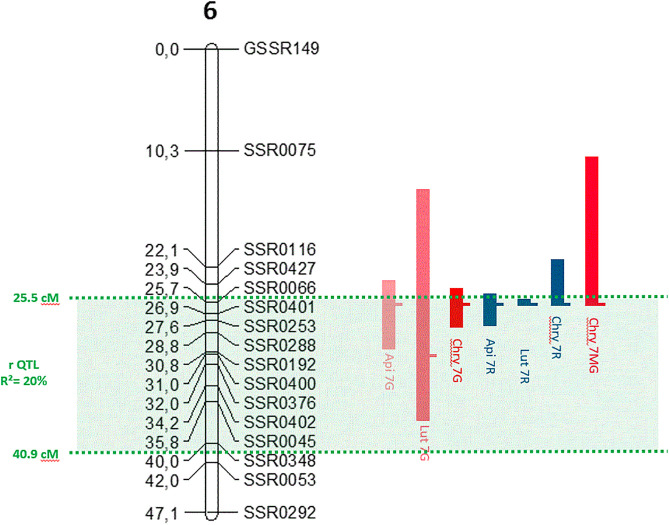
Flavonoid mQTLs co-localizing with the resistance rQTL on carrot chromosome 6. Flavonoid abbreviations are Api for Apigenin, Lut for Luteolin and Chry for Chrysoeriol; 7G for 7-*O* glucoside, 7R for 7-*O* rutinoside, and 7MG for 7-*O* malonylglucoside. Flavonoid mQTLs are represented by red blocks when they are positively linked to disease severity or blue blocks when negatively linked. Blocks length and position indicate the 1 LOD support interval with the maximum peak position indicated by a small lateral horizontal bar. SSR codes on the right of the chr6 are microsatellite markers with position indicated in centimorgans on the left as published by Koutouan *et al*. [9]. The rQTL described as RL14 on chr6 by these authors is indicated in green. R² = Explained resistance variation; cM = centimorgan.

Interestingly, most of the mQTLs associated with these seven metabolites co-localized with the major rQTL on chr6 (R² = 20%) (**Fig 2**). The seven mQTLs overlapped and most of them had their maximum positions at 25.8 cM. The Lut 7G mQTL had his peak at 31.1 cM.

### Identification of candidate genes

**Fig 3:**
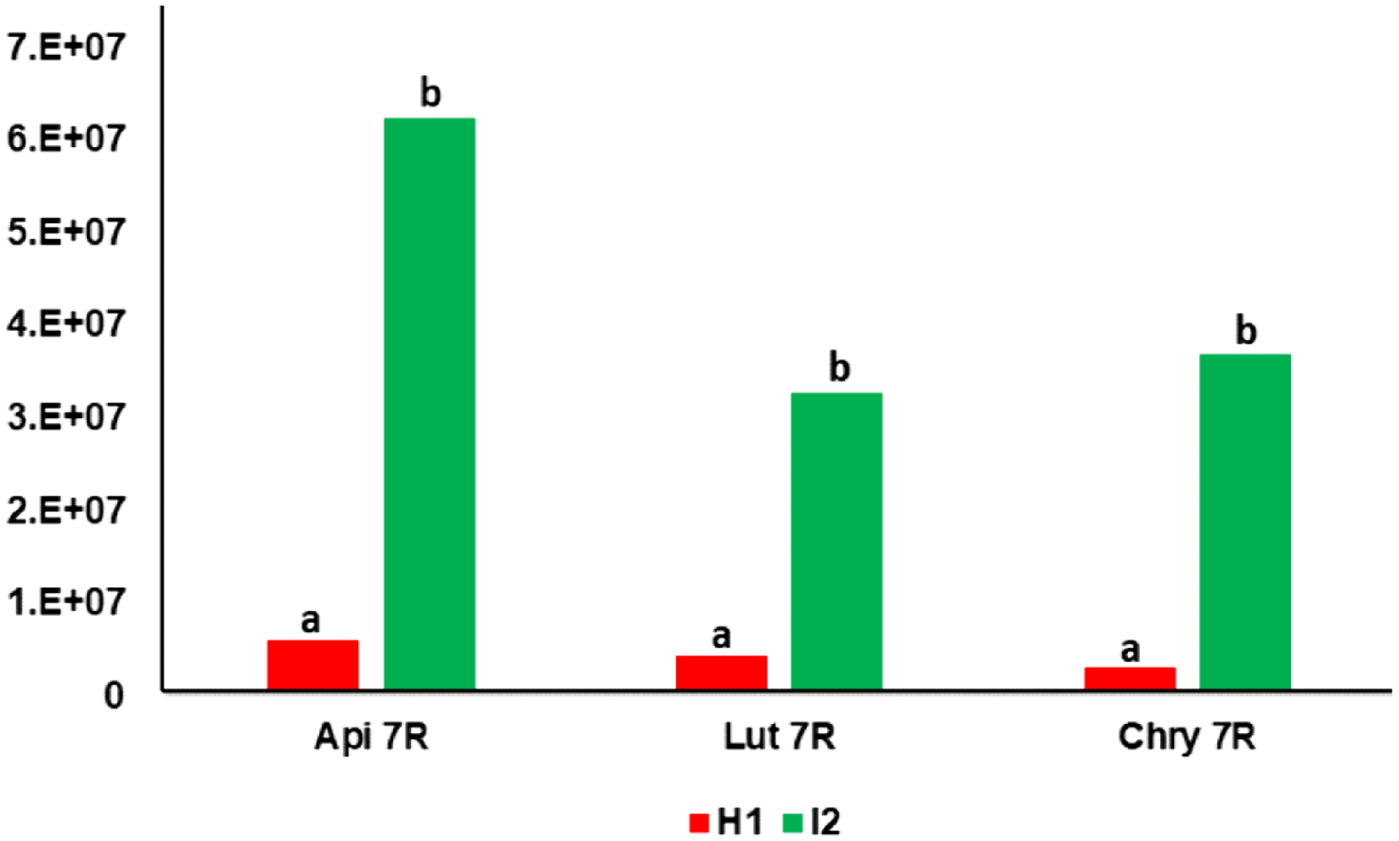
Accumulation of apigenin-7-*O*-rutinoside (Api 7R), luteolin-7-*O*-rutinoside (Lut 7R), and chrysoeriol-7-*O*-rutinoside (Chry 7R) in the leaves of the two carrot genotypes H1 and I2. The red bars correspond to the susceptible genotype H1, the green bars to the partially resistant genotype I2. The Y-axis represents peak area values from mass-spectrometry-based quantification (arbitrary unit). Data were obtained from four biological replicates. Letters denote group differences based on Tukey’s tests for each of the three metabolites with error threshold = 0.05 (S1 File).

Prior to gene expression analyses, we demonstrated that the differences between the susceptible genotype (H1) and the resistant genotype (I2) in the content of metabolites associated with resistance were highly significant (**Fig 3**), which is consistent with previous observations.

**Fig 4:**
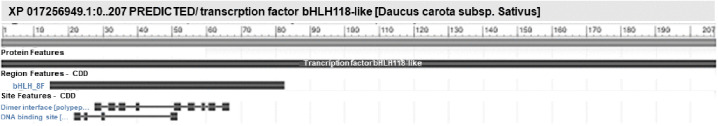
Conserved domains of DcbHLH162-like protein sequence (NCBI).

In order to focus on the search for candidate genes within the mQTL-rQTL co-localization region on chr6, we performed an *in silico* analysis of the genomic interval flanked by SSR0066 and SSR0292. Subsequently, a microarray analysis comparing the transcriptomes of H1 and I2 was carried out to identify, among the 292 genes located within this interval, those that are significantly overexpressed in I2 compared to H1 and potentially involved in the biosynthesis of the three flavone-rutinosides. These combined analyses have notably highlighted the *LOC108226497* (https://www.ncbi.nlm.nih.gov/gene/LOC108226497), predicted to encode a basic helix-loop-helix transcription factor, more specifically a bHLH162-like protein composed of 207 amino acids. This protein possesses a highly conserved bHLH domain spanning 82 residues from its N-terminal side (NCBI). The domain includes a six-residue DNA binding site and a 16-residue dimerization interface (**Fig 4**).

### Validation of bHLH by transformation

**Fig 5:**
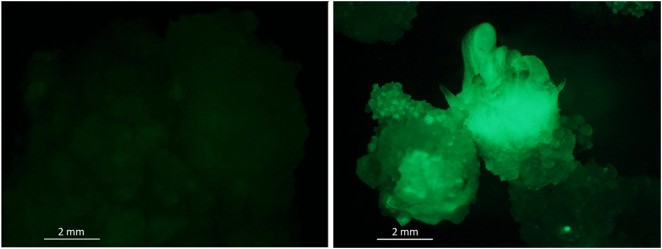
Fluorescence of the GFP in calli of non-transformed carrot genotype H1 (left), compared to H1 bHLH calli (right) harboring the *DcbHLH162-like* gene (*LOC108226497)* under the control of the *CaMV 35S* promoter.

Selection of potentially transformed calli exposed to *A. tumefaciens* was based on their ability to develop on kanamycin-supplemented medium and the observation of a GFP fluorescence signal (**Fig 5**). Transformants with empty plasmid will be further called H1 EV for empty vector. Transformants harboring the *LOC108226497* under the control of the *CaMV 35S* promoter, allowing a constitutive overexpression of the candidate gene, will be called H1 bHLH over-expressors. Non-transformed calli will be referred to as NT. The *DcbHLH162-like* gene will be simply called bHLH.

**Fig 6:**
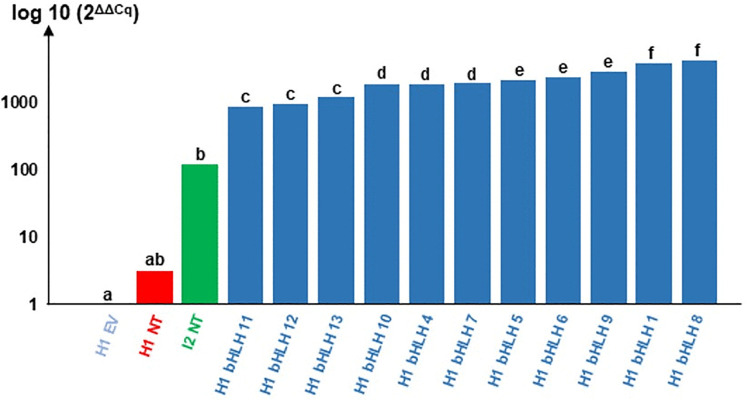
Expression level of the *DcbHLH162-like* gene in calli of 11 different transformation events (called H1 bHLH 1 to 13) compared to non-transformed controls (NT) and empty vector transformants (EV). H1 NT: native carrot genotype susceptible to ALB (represented in red); I2 NT: native partially resistant carrot genotype (represented in green). H1 bHLH 1 to 13 (represented in dark blue) are transformants harboring the *LOC108226497* under the control of the *CaMV 35S* promoter. Y-axis indicates the expression level of the target gene in the calli (log scale), referring to its mean expression level in 7 events of H1 empty vector modality (represented in blue) and calculated with 2^ΔΔCq^ method [42]. Data were obtained from three technical replicates in a qPCR run. Letters denote group differences based on Conover’s all-pairs test with error threshold = 0.05 (S2 File).

Gene expression was measured in calli derived from 11 different transformation events (H1 bHLH 1–13), 2 non-transformed controls (H1 NT and I2 NT), and the reference empty vector transformants (EV) using the primers BHLH-F 5’- ATATTTCGCGTCTCCTTTTATGC-3’ and BHLH-R 5’- GCCGATCCCTCCTGCAAT-3’ (**Fig 6**). As expected, the expression of *DcbHLH162-like* gene in the H1 NT control was very similar to that in H1 EV. Expression in I2 NT was 119 times higher than in H1 EV, and 40 times higher than in H1 NT. For each of the bHLH putative over-expressors, the gene expression level was significantly higher than in H1 EV (up to 4134 times higher for H1 bHLH 8). This demonstrates the high success of the transformation process.

**Fig 7:**
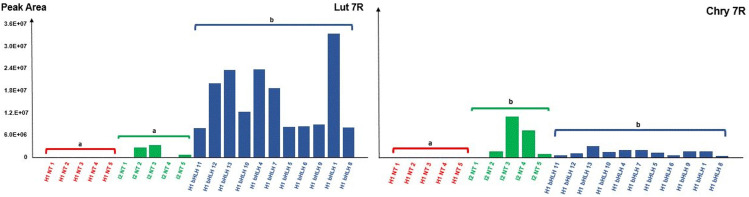
Accumulation of luteolin-7-*O*-rutinoside (Lut 7R), and chrysoeriol-7-*O*-rutinoside (Chry 7R) in the calli of the two native or transformed carrot genotypes H1 and I2. H1 NT 1 to 5 are non-transformed H1 calli; I2 NT 1 to 5 are non-transformed I2 calli. H1 bHLH 1 to 13 are 11 independent transformation events of H1 transformants harboring the *DcbHLH162-like* gene (*LOC108226497*) under the control of the *CaMV 35S* promoter. Y-axis represents peak area values from mass-spectrometry-based quantification (arbitrary unit). For each of the two metabolites, letters a and b indicate significant group differences based on Pairwise comparisons between the three groups H1 NT, I2 NT and H1 bHLH, the different calli within a group (1 to 5 for H1 NT and I2 NT, or 1 to 13 for H1 bHLH) being considered as biological replicates. This analysis was based on Conover’s all-pairs tests. (**S3** File).

Regarding the metabolic phenotypes (**Fig 7**, **S3** File), Lut 7R was the most abundant metabolite in bHLH over-expressors calli, followed by Chry 7R. In both cases, these levels in bHLH over-expressors calli were significantly different from those in H1 NT samples. Although Lut 7R levels exhibited a tendency to be higher in I2 NT samples – approximately 100 fold – compared to H1 NT samples, the difference was not statistically significant. In contrast, Chry 7R levels in I2 NT samples clustered with those of H1 bHLH over-expressors and were significantly different from H1 NT samples. Api 7R was not detected in any of the samples.

### Biological activity of callus extracts

According to material availability, calli from events n° 4, 7 and 13 were selected to represent bHLH over-expressors, while calli called H1 NT 0, 1, 2 and I2 NT 2, 3 were selected to represent non-transformed H1 and I2 respectively.

**Fig 8:**
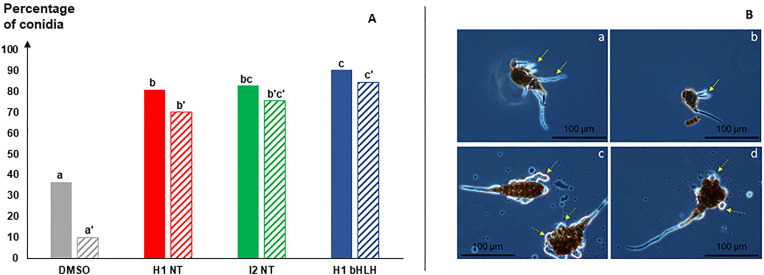
Effect of transformed callus extracts on *A. dauci* condia germination. A, Germination (solid bars) and disruption (hatched bars) percentages calculated from 3200 microscope observations of conidia exposed to callus extracts or control solution (1% DMSO) (400 conidia per modality). Germination statistics: ANOVA p ≤ 0.001***; disruption statistics: ANOVA p ≤ 0.001***. Letters a, b and c displayed group significance from Tukey’s multiple comparison test for germination rates. Letters a’, b’ and c’ displayed group significance from Tukey’s multiple comparison test for disruption rates. Error threshold = 0.05 (**S4** File**)**. B, pictures of normal (plain arrows on a and b) or disrupted (dashed arrows on c and d) hyphae.

When exposed to callus extracts, the germination rate of *A. dauci* conidia tended to increase, regardless of the callus type, compared to the control DMSO **(Fig 8A**, **solid bars)**. This stimulation was significantly higher with the H1 bHLH callus extract than with the non-transformed one (H1 NT). Non-transformed I2 extract exhibited an intermediate effect between the two. However, particularly with the H1 bHLH extract, nearly all emerging hyphae were disrupted (**Fig 8A, dashed bars; Fig 8B, c and d)**. This disruption rate was significantly higher with H1 bHLH extract than with H1 NT extract and was markedly higher than under the control condition (1% DMSO). Again, the I2 extract showed an intermediate effect between the H1 NT and H1 bHLH extracts (**S4** File).

## Discussion

Effective and sustainable management of ALB is essential to ensure stable carrot yields. The development of elite cultivars with durable and increased resistance is a key objective for reducing fungicide applications.

In previous work, we identified terpenes and flavonoids associated with resistance to ALB in carrot [16]. In the present study, as a complement to the work on terpene-related resistance [9], we investigated potential flavonoid-based resistance mechanisms to ALB in carrot. Our results proved that seven of the twelve flavonoids previously highlighted [16) were significantly associated with resistance, either positively – Api 7R, Lut 7R, and Chry 7R — or negatively —Api 7G, Lut 7G, Chry 7G and Chry 7MG.

In a recent paper, based on a positive association between Api 7R, Lut 7R, and Chry 7R accumulation and resistance, we proposed using these three metabolites as biomarkers of resistance in breeding programs. This previous study was performed on genetically unrelated genotypes, i.e., without any known genealogical relationship [27]. In the present work, the studied genotypes all originate from a cross between H1 and I2, susceptible and resistant genotypes respectively, and belong to a population highly segregating for resistance. Since resistance levels in the progeny remain strongly associated with the accumulation of these three metabolites, this further supports their relevance as biomarkers.

We not only confirmed the relationship between flavonoid content and resistance level, but also mapped the QTLs underlying the accumulation of the seven metabolites and demonstrated their co-localization with resistance QTLs. Even more interestingly, their variation is primarily explained by mQTLs located on chromosome 6, in close proximity to the major RL14 rQTL, the elucidation of which was of particular interest. Moreover, the alleles associated with their higher accumulation, originated from the resistant parent I2. These observations logically support the hypothesis that in addition to terpenes previously highlighted [9], Api 7R, Lut 7R, and Chry 7R may play a direct role in resistance to ALB. In fact, studies on other plant pathogen systems support our findings on flavonoids as resistance factors.

Bollina *et al*. [24] showed that flavonoids such as kaempferol and naringenin were associated with resistance of barley cultivars against FHB. Notably, naringenin is a precursor of apigenin [43]. [25] reported that Api 7R is a constitutive metabolite linked to resistance against FHB in wheat.

Tomić *et al*. [44] identified Lut 7R as an important component of a methanol extract from *Athamanta turbith* subsp. *haynaldii*, an Apiaceae species. This methanol extract inhibited the growth of *Candida albicans in vitro*. Api 7R and Lut 7R were found to inhibit the growth of the phytopathogenic fungi *Aspergillus niger* and *Cladosporium cucumerinum* [26], Lut 7R showing the highest effect.

The other four metabolites significantly associated with resistance—but negatively—are glucoside compounds that may potentially compete with the first three. Indeed, all of them belong to the same biosynthesis pathway. The substrate 7G can either accumulate in its original form or be converted into 7MG or 7R. It is the 7R conversions of glucosides into rutinosides (Api 7R, Lut 7R, and Chry 7R) that were preferentially activated in resistant individuals. Future investigations could focus on analysing the ratios between the different metabolites to assess whether such competition plays a critical role. If confirmed, breeders will have to screen materials with the favourable balance between 7R and 7G or 7MG to obtain breeding lines that are more resistant.

Based on these contrasting results observed between rutinosides and glucosides, we propose that a balance may exist between “resistance” and “susceptibility” branches of the flavone biosynthesis pathway, determined by their glycosylation patterns. This biosynthetic step may be catalyzed by an enzyme with rhamnosyltransferase activity [16]. We therefore searched for genes differentially expressed between H1 and I2, underlying the co-localization region, and potentially associated with this balance.

A gene encoding a bHLH transcription factor was identified within the co-localization region of chr6. Its structural characterization revealed a DNA-binding site comprising six residues, likely recognizing E-box consensus sequences in various gene promoters, as well as a dimer interface of 16 residues where polypeptide binding is expected to occur. Heterodimerization is particularly noteworthy, as the bHLH transcription factor may form a complex with other transcription factors such as R2R3-MYB and WD40, to assemble a MBW complex capable of regulating the flavonoid biosynthesis and accumulation, as already documented in other plant species [45–47], including carrot [48,49]. Based on these findings, we hypothesize that the bHLH gene identified in our study may play a role in regulating flavonoid associated with resistance to ALB.

In the literature, flavonoid biosynthesis has also been shown to be regulated by the MBW complex, which involves interactions between R2R3-MYB, bHLH, and WD40-type transcription factors [17,39–41,50–52]. For instance, overexpressing of *TCP3* (a member of the bHLH family) in transgenic *Arabidopsis* plants resulted in the hyper-accumulation of flavanols, anthocyanins and proanthocyanidins [53]. Conversely, Burr *et al*. [54] reported that *Intensifier 1* (a bHLH transcription factor) from *Zea mays* negatively regulates anthocyanins accumulation. Similarly, Dhokane *et al*. [55] identified a gene encoding a bHLH transcription factor within the *QRL-Fhb2* region, which is associated with wheat resistance against FHB. The expression of this gene was significantly higher in resistant RILs compared to susceptible ones. They also observed a high constitutive accumulation of flavonoids in resistant RILs.

To assess the potential role of this gene in carrot resistance to ALB, we successfully transformed the susceptible genotype H1, which naturally accumulate low levels of the candidate flavones compared to the resistant genotype I2, with the *DcbHLH162-like* gene (*LOC108226497),* under the control of the *CaMV 35S* promoter. The gene was successfully overexpressed in all selected H1 transformed calli, leading to higher expression levels than in H1 NT and even more than in I2 NT calli. The latter result was probably due to the strong activity of the *CaMV 35S* promoter.

The regeneration of bHLH162-like transgenic whole plants and the characterization of their offspring will take several years. As an alternative, in order to validate the *bHLH162-like* gene and associated flavonoids as resistance factors to ALB in carrot, we proposed an initial functional characterization through metabolic and transcriptomic analysis of calli, followed by the evaluation of callus extract effects on *A. dauci* conidia behaviors.

The metabolic characterization of the callus extracts was consistent with expectations, revealing a significant quantitative increase in Lut 7R and Chry 7R in bHLH-transformed H1 calli compared to non-transformed H1 calli. Non-transformed I2 calli displayed an intermediate profile, clustering with H1 NT calli for Lut 7R levels, and with H1 bHLH calli for Chry 7R. Based on previous quantifications conducted on whole plants, the accumulation of Api 7R in calli could have been expected. However, the absence of Api 7R detection is consistent with prior unpublished studies in which attempts to detect Api 7R at the callus stage were unsuccessful. A plausible explanation for the lack of Api 7R accumulation could be an elevated activity of the enzyme EC 1.14.13.21 in calli compared to leaves. This flavonoid 3’-hydroxylase enzyme was described by Koutouan *et al*. [16] as responsible for the hydroxylation of agenin to luteolin.

The biological activity of extracts from H1 NT and transformed H1 calli indicate that contact between the carrot matrix and the conidia may serve as a clear signal for conidia germination. Indeed, conidia germination rates in the presence of callus extracts were significantly and strongly increased compared to the DMSO culture medium. This effect was statistically more pronounced in H1 bHLH than in H1 NT callus extracts. This suggests that the stimulating compounds are more abundant in the transformants than in the non-transformed calli. I2 also tends to stimulate conidial germination more than H1 NT even if differences were not statistically significant. This “stimulation” may seem surprising. However, it is interesting to link these observations to the findings of Boedo *et al.* [56], who reported that *A. dauci* conidia produced significantly more germ tubes when inoculated on Bolero leaves – an ALB partially resistant variety – than on Presto leaves, a susceptible variety, despite the fact that disease progression was much more slower on Bolero. It is not unlikely that flavonoids are responsible for this phenomenon. Indeed, stimulation of fungal growth by flavonoid glycosides has been previously reported, primarily in the context of the beneficial plant-fungus interactions [57]. However, Ruan *et al.* [58], in the context of pathogenic interactions, also found that apigenin-7-O-glucoside (Api 7G) and pisatin from pea (*Pisum sativum*) root exudates stimulated the germination of *Fusarium solani* f. sp. *pisi* spores. The stimulation of pathogen development by defence-related flavonoids has been interpreted as an adaptation of the pathogen to its host, as summarised by Straney *et al*. [59]. To understand how this stimulation may result in resistance, it is important to analyse hyphae growth.

Conidia exposed to bHLH calli extracts exhibited a very high germination rate. However, the vast majority of emitted hyphae did not survive and underwent lysis. This phenomenon probably compromised the pathogenic potential of these hyphae. I2 NT showed intermediate fungitoxic properties between H1 NT and H1 bHLH callus extracts, in agreement with their intermediate flavonoid content.

The three compounds associated with resistance are biochemically linked, with apigenin being the precursor of luteolin, and luteolin being the precursor of chrysoeriol [16,43]. An hypothesis of a multi-step co-evolution between the plant and the pathogenic fungus could be proposed as follows. Initially, Api 7R-containing carrot resisted to *A. dauci* until the fungus adapted itself to Api 7R and used this molecule to recognize the host. In a second step, carrot evolved to transform Api 7R into Lut 7R, that was efficient until a new fungal adaptation. In a third step, carrot evolved to produce a more complex molecule from Lut 7R, namely Chry 7R.

In such a scenario, Api 7R and Lut 7R conferred resistance to the carrot plant in the past, but they would now be overcome by the pathogen, which uses them as signalling molecules indicating host presence. Fungal pathogens evading flavonoid-based host defence have already been reported, for example by detoxifying active compounds from the host plant or by secreting catabolic enzymes [60]. In the case of *Sclerotinia sclerotiorum*, its virulence against *Arabidopsis thaliana* was associated with its ability to metabolize rutin, by releasing the rutinose moiety, prior to degradation of the aglycone by a quercetin 2,3-dioxygenase [61]. In tomato (*Solanum lycopersicum*)-*Cladosporium fulvum* interaction, the increased pathogen virulence was conferred by a tomatinase enzyme, capable of degrading α-tomatin, a saponin with antimicrobial activity in plants [62]. Similar mechanisms, yet to be discovered, may exist in *A. dauci*.

Chry 7R, on the other hand, would remain an effective weapon to eliminate the pathogen by disrupting its hyphae. Direct antifungal activities of flavonoids have already been documented and summarized by Ramaroson *et al*. [17], Das *et al*. [22] or Patil *et al.* [63]. Effects such as fungal cell wall invagination, shrinkage, and intracellular calcium and potassium leakage leading to osmotic imbalance have been described for example in *Candida albicans* [64]. A putative antifungal activity of Chry 7R may therefore be further investigated.

From an evolutionary perspective, the addition of another step in the flavone pathway – leading to increased structural complexity from apigenin to luteolin and then to chrysoeriol – could reflect an adaptative response to a changing environment. More specifically, the biosynthesis of chrysoeriol from luteolin may indicate a renewed need to fully control the pathogen, following the partial loss of protection conferred by Lut 7R alone. This interpretation is supported by the observed dual role of callus extracts: they not only stimulated hyphal emission – possibly due to Lut 7R – but also induced a strong hyphal disruption, likely attributable to chry 7R. This resulted almost in a complete loss of pathogenicity, with 85% of hyphae disrupted in H1 bHLH modality compared to only 10% in the control. Considering previous data and the observed loss of pathogenicity, bHLH is further supported as being implicated in *A. dauci* resistance, likely through the three flavonoids biosynthesis.

## Supporting information

S1 File Statistical analyses of apigenin-7-*O*-rutinoside, luteolin-7-*O*-rutinoside, and chrysoeriol-7-*O*-rutinoside accumulations in the leaves of the two carrot genotypes. (PDF)

S2 File Statistical analyses of Expression levels of the DcbHLH162-like gene in calli of 11 different H1 transformation events. (PDF)

S3 File Statistical analyses of accumulation levels of luteolin-7-O-rutinoside, and chrysoeriol-7-O-rutinoside in the calli of the two native or transformed carrot genotypes H1 and I2. (PDF)

S4 File Statistical analyses of germination and disruption percentages calculated from 3200 microscope observations of conidia exposed to callus extracts or control solution. (PDF)

## References

[1] Nunez JJ, Davis RM. Diseases of carrot. 2016. https://www.apsnet.org/edcenter/resources/commonnames/Pages/Carrot.aspx

[2] Geoffriau E, Simon PW. Carrot and related Apiaceae crops. 2 ed. Wallingford, Oxfordshire, UK: CABI; 2020.

[3] Souza RT, Forcelini CA, Reis EM, Calvete EO. Freqüência de Alternaria dauci E Cercospora carotae como agentes da queima das folhas da cenoura em Passo Fundo, RS. Fitopatol bras. 2001;26(3):614–8. doi: 10.1590/s0100-41582001000300006

[4] Farrar JJ, Pryor BM, Davis RM. Alternaria diseases of carrot. Plant Dis. 2004;88(8):776–84. 30812503 10.1094/PDIS.2004.88.8.776

[5] Boedo C, Le Clerc V, Briard M, Simoneau P, Chevalier M, Georgeault S, et al. Impact of carrot resistance on development of the Alternaria leaf blight pathogen (Alternaria dauci). Eur J Plant Pathol. 2008;121(1):55–66. doi: 10.1007/s10658-007-9241-6

[6] Le Clerc V, Briard M. Carrot disease management. In: Carrots and related Apiaceae crops. 2nd ed. Wallingford, Oxfordshire, UK: CABI; 2020. 115–29.

[7] Le Clerc V, Suel A, Pawelec A, Marques S, Huet S, Lecomte M, et al. Is there variety × isolate interaction in the polygenic quantitative resistance of carrot to *Alternaria dauci*? Euphytica. 2015;202(2):235–43.

[8] Le Clerc V, Aubert C, Cottet V, Yovanopoulos C, Piquet M, Suel A, et al. Breeding for carrot resistance to Alternaria dauci without compromising taste. Mol Breed. 2019;39(4):59.

[9] Koutouan CE, Le Clerc V, Suel A, Hamama L, Claudel P, Halter D, et al. Co-localization of resistance and metabolic quantitative trait loci on carrot genome reveals fungitoxic terpenes and related candidate genes associated with the resistance to Alternaria dauci. Metabolites. 2023;13(1):71. doi: 10.3390/metabo13010071 36676996 PMC9863879

[10] Le Clerc V, Pawelec A, Birolleau-Touchard C, Suel A, Briard M. Genetic architecture of factors underlying partial resistance to Alternaria leaf blight in carrot. Theor Appl Genet. 2009;118(7):1251–9. doi: 10.1007/s00122-009-0978-5 19214391

[11] Le Clerc V, Marques S, Suel A, Huet S, Hamama L, Voisine L, et al. QTL mapping of carrot resistance to leaf blight with connected populations: stability across years and consequences for breeding. Theor Appl Genet. 2015;128(11):2177–87. doi: 10.1007/s00122-015-2576-z 26152576

[12] Poland JA, Balint-Kurti PJ, Wisser RJ, Pratt RC, Nelson RJ. Shades of gray: the world of quantitative disease resistance. Trends Plant Sci. 2009;14(1):21–9. doi: 10.1016/j.tplants.2008.10.006 19062327

[13] Rowe HC, Kliebenstein DJ. Complex genetics control natural variation in Arabidopsis thaliana resistance to Botrytis cinerea. Genetics. 2008;180(4):2237–50. doi: 10.1534/genetics.108.091439 18845849 PMC2600955

[14] Hamzehzarghani H, Paranidharan V, Abu-Nada Y, Kushalappa AC, Mamer O, Somers D. Metabolic profiling to discriminate wheat near isogenic lines, with quantitative trait loci at chromosome 2DL, varying in resistance to Fusarium head blight. Can J Plant Sci. 2008;88(4):789–97. doi: 10.4141/cjps07209

[15] Gunnaiah R, Kushalappa AC, Duggavathi R, Fox S, Somers DJ. Integrated metabolo-proteomic approach to decipher the mechanisms by which wheat QTL (Fhb1) contributes to resistance against Fusarium graminearum. PLoS One. 2012;7(7):e40695. doi: 10.1371/journal.pone.0040695 22866179 PMC3398977

[16] Koutouan C, Clerc VL, Baltenweck R, Claudel P, Halter D, Hugueney P, et al. Link between carrot leaf secondary metabolites and resistance to Alternaria dauci. Sci Rep. 2018;8(1):13746. doi: 10.1038/s41598-018-31700-2 30213972 PMC6137067

[17] Ramaroson M-L, Koutouan C, Helesbeux J-J, Le Clerc V, Hamama L, Geoffriau E, et al. Role of phenylpropanoids and flavonoids in plant resistance to pests and diseases. Molecules. 2022;27(23):8371. doi: 10.3390/molecules27238371 36500459 PMC9735708

[18] Shirley BW. Flavonoids in seeds and grains: physiological function, agronomic importance and the genetics of biosynthesis. Seed Sci Res. 1998;8(4):415–22. doi: 10.1017/s0960258500004372

[19] Winkel-Shirley B. Flavonoid biosynthesis. A colorful model for genetics, biochemistry, cell biology, and biotechnology. Plant Physiol. 2001;126(2):485–93. doi: 10.1104/pp.126.2.485 11402179 PMC1540115

[20] Falcone Ferreyra ML, Rius SP, Casati P. Flavonoids: biosynthesis, biological functions, and biotechnological applications. Front Plant Sci. 2012;3:222. doi: 10.3389/fpls.2012.00222 23060891 PMC3460232

[21] Kumar GA, Kumar S, Bhardwaj R, Swapnil P, Meena M, Seth CS, et al. Recent advancements in multifaceted roles of flavonoids in plant-rhizomicrobiome interactions. Front Plant Sci. 2024;14:1297706. doi: 10.3389/fpls.2023.1297706 38250451 PMC10796613

[22] Das A, Choudhury S, Gopinath V, Majeed W, Chakraborty S, Bhairavi KS, et al. Functions of flavonoids in plant, pathogen, and opportunistic fungal interactions. In: Opportunistic fungi, nematode and plant interactions. Springer Nature Singapore; 2024. 91–123. doi: 10.1007/978-981-97-2045-3_6

[23] Soleimani H, Mostowfizadeh-Ghalamfarsa R, Ghanadian SM. Celery flavonoid-rich extract significantly reduces cucumber powdery mildew severity and enhances plant defense responses. Sci Rep. 2025;15(1):10589. doi: 10.1038/s41598-025-95365-4 40148466 PMC11950358

[24] Bollina V, Kumaraswamy GK, Kushalappa AC, Choo TM, Dion Y, Rioux S, et al. Mass spectrometry-based metabolomics application to identify quantitative resistance-related metabolites in barley against Fusarium head blight. Mol Plant Pathol. 2010;11(6):769–82. doi: 10.1111/j.1364-3703.2010.00643.x 21029322 PMC6640360

[25] Gunnaiah R, Kushalappa AC. Metabolomics deciphers the host resistance mechanisms in wheat cultivar Sumai-3, against trichothecene producing and non-producing isolates of Fusarium graminearum. Plant Physiol Biochem. 2014;83:40–50. doi: 10.1016/j.plaphy.2014.07.002 25084325

[26] Zhu X, Zhang H, Wang T. Phenolic compounds from the leaf extract of artichoke (Cynara scolymus L.) and their antimicrobial activities. J Chem Eng Data. 2004;54(3):945–9. 10.1021/jf0490192 15563206

[27] Ramaroson ML, Koutouan CE, Ghaziri AE, Baltenweck R, Claudel P, Hugueney P, et al. Flavonoid compounds as a way to identify sources of carrot resistance to Alternaria leaf blight. Mol Breed. 2025;45(6):55. doi: 10.1007/s11032-025-01573-1 40520328 PMC12167411

[28] Pawelec A, Dubourg C, Briard M. Evaluation of carrot resistance to alternaria leaf blight in controlled environments. Plant Pathology. 2006;55(1):68–72. doi: 10.1111/j.1365-3059.2006.01290.x

[29] Boedo C, Benichou S, Berruyer R, Bersihand S, Dongo A, Simoneau P, et al. Evaluating aggressiveness and host range of Alternaria dauci in a controlled environment. Plant Pathology. 2011;61(1):63–75. doi: 10.1111/j.1365-3059.2011.02494.x

[30] Rstudio T. RStudio: integrated development for R. Boston, MA. 2016.

[31] van den Berg RA, Hoefsloot HCJ, Westerhuis JA, Smilde AK, van der Werf MJ. Centering, scaling, and transformations: improving the biological information content of metabolomics data. BMC Genomics. 2006;7:142. doi: 10.1186/1471-2164-7-142 16762068 PMC1534033

[32] Jourjon M-F, Jasson S, Marcel J, Ngom B, Mangin B. MCQTL: multi-allelic QTL mapping in multi-cross design. Bioinformatics. 2005;21(1):128–30. doi: 10.1093/bioinformatics/bth481 15319261

[33] Iorizzo M, Ellison S, Senalik D, Zeng P, Satapoomin P, Huang J, et al. A high-quality carrot genome assembly provides new insights into carotenoid accumulation and asterid genome evolution. Nat Genet. 2016;48(6):657–66. doi: 10.1038/ng.3565 27158781

[34] Kearse M, Moir R, Wilson A, Stones-Havas S, Cheung M, Sturrock S, et al. Geneious basic: an integrated and extendable desktop software platform for the organization and analysis of sequence data. Bioinformatics. 2012;28(12):1647–9. doi: 10.1093/bioinformatics/bts199 22543367 PMC3371832

[35] RNA isolation user manual NucleoSpin® RNA Plus. Macherey-Nagel; 2016.

[36] Agilent Technologies inc. Two-color microarray-based gene expression analysis (low input quick amp labeling) protocol. 2015. https://www.agilent.com/cs/library/usermanuals/public/G4140-90050_GeneExpression_TwoColor_6.9.pdf

[37] Gagnot S, Tamby J-P, Martin-Magniette M-L, Bitton F, Taconnat L, Balzergue S, et al. CATdb: a public access to Arabidopsis transcriptome data from the URGV-CATMA platform. Nucleic Acids Res. 2008;36(Database issue):D986-90. doi: 10.1093/nar/gkm757 17940091 PMC2238931

[38] Lecomte M, Hamama L, Voisine L, Gatto J, Hélesbeux J-J, Séraphin D, et al. Partial resistance of carrot to Alternaria dauci correlates with in vitro cultured carrot cell resistance to fungal exudates. PLoS One. 2014;9(7):e101008. doi: 10.1371/journal.pone.0101008 24983469 PMC4077726

[39] Courtial J, Hamama L, Helesbeux J-J, Lecomte M, Renaux Y, Guichard E, et al. Aldaulactone - an original phytotoxic secondary metabolite involved in the aggressiveness of Alternaria dauci on carrot. Front Plant Sci. 2018;9:502. doi: 10.3389/fpls.2018.00502 29774035 PMC5943595

[40] Karimi M, Inzé D, Depicker A. GATEWAY vectors for Agrobacterium-mediated plant transformation. Trends Plant Sci. 2002;7(5):193–5. doi: 10.1016/s1360-1385(02)02251-3 11992820

[41] Hamama L, Bosselut J, Voisine L, Thouroude T, Ogé L, Chameau J, et al. Rose FT homologous gene overexpression affects flowering and vegetative development behavior in two different rose genotypes. Plant Cell Tiss Organ Cult. 2024;156(3). doi: 10.1007/s11240-024-02695-8

[42] Livak KJ, Schmittgen TD. Analysis of relative gene expression data using real-time quantitative PCR and the 2(-Delta Delta C(T)) Method. Methods. 2001;25(4):402–8. doi: 10.1006/meth.2001.1262 11846609

[43] Kanehisa M, Furumichi M, Tanabe M, Sato Y, Morishima K. KEGG: new perspectives on genomes, pathways, diseases and drugs. Nucleic Acids Res. 2017;45(D1):D353–61. doi: 10.1093/nar/gkw1092 27899662 PMC5210567

[44] Tomić A, Petrović S, Pavlović M, Trajkovski B, Milenković M, Vučićević D. Antimicrobial and antioxidant properties of methanol extracts of two Athamanta turbith subspecies. Pharm Biol. 2009;47(4):314–9.

[45] Zhao F, Li G, Hu P, Zhao X, Li L, Wei W, et al. Identification of basic/helix-loop-helix transcription factors reveals candidate genes involved in anthocyanin biosynthesis from the strawberry white-flesh mutant. Sci Rep. 2018;8(1):2721. doi: 10.1038/s41598-018-21136-z 29426907 PMC5807450

[46] Guo J, Wu Y, Wang G, Wang T, Cao F. Integrated analysis of the transcriptome and metabolome in young and mature leaves of Ginkgo biloba L. Ind Crop Prod. 2020;143:111906. doi: 10.1016/j.indcrop.2019.111906

[47] Xu Y, Ge X, Lv Y, Yang Z, Li F, Yang Z. Engineering plant hosts for high-efficiency accumulation of flavonoids: advances, challenges and perspectives. Biotechnol Adv. 2025;84:108692. doi: 10.1016/j.biotechadv.2025.108692 40850536

[48] Kodama M, Brinch-Pedersen H, Sharma S, Holme IB, Joernsgaard B, Dzhanfezova T, et al. Identification of transcription factor genes involved in anthocyanin biosynthesis in carrot (Daucus carota L.) using RNA-Seq. BMC Genomics. 2018;19(1):811. doi: 10.1186/s12864-018-5135-6 30409110 PMC6225646

[49] Meng G, Clausen SK, Rasmussen SK. Transcriptome analysis reveals candidate genes related to Anthocyanin biosynthesis in different carrot genotypes and tissues. Plants (Basel). 2020;9(3):344. doi: 10.3390/plants9030344 32182858 PMC7154819

[50] Hichri I, Heppel SC, Pillet J, Léon C, Czemmel S, Delrot S, et al. The basic helix-loop-helix transcription factor MYC1 is involved in the regulation of the flavonoid biosynthesis pathway in grapevine. Mol Plant. 2010;3(3):509–23. doi: 10.1093/mp/ssp118 20118183

[51] Hichri I, Barrieu F, Bogs J, Kappel C, Delrot S, Lauvergeat V. Recent advances in the transcriptional regulation of the flavonoid biosynthetic pathway. J Exp Bot. 2011;62(8):2465–83. doi: 10.1093/jxb/erq442 21278228

[52] Xu W, Dubos C, Lepiniec L. Transcriptional control of flavonoid biosynthesis by MYB-bHLH-WDR complexes. Trends Plant Sci. 2015;20(3):176–85. doi: 10.1016/j.tplants.2014.12.001 25577424

[53] Li S, Zachgo S. TCP3 interacts with R2R3-MYB proteins, promotes flavonoid biosynthesis and negatively regulates the auxin response in Arabidopsis thaliana. Plant J. 2013;76(6):901–13. doi: 10.1111/tpj.12348 24118612

[54] Burr FA, Burr B, Scheffler BE, Blewitt M, Wienand U, Matz EC. The maize repressor-like gene intensifier1 shares homology with the r1/b1 multigene family of transcription factors and exhibits missplicing. Plant Cell. 1996;8(8):1249–59. doi: 10.1105/tpc.8.8.1249 8776895 PMC161237

[55] Dhokane D, Karre S, Kushalappa AC, McCartney C. Integrated metabolo-transcriptomics reveals fusarium head blight candidate resistance genes in wheat QTL-Fhb2. PLoS One. 2016;11(5):e0155851. doi: 10.1371/journal.pone.0155851 27232496 PMC4883744

[56] Boedo C, Berruyer R, Lecomte M, Bersihand S, Briard M, Le Clerc V, et al. Evaluation of different methods for the characterization of carrot resistance to the alternaria leaf blight pathogen ( Alternaria dauci ) revealed two qualitatively different resistances. Plant Pathol. 2010;59(2):368–75. doi: 10.1111/j.1365-3059.2009.02218.x

[57] Lagrange H, Jay-Allgmand C, Lapeyrie F. Rutin, the phenolglycoside from eucalyptus root exudates, stimulates Pisolithus hyphal growth at picomolar concentrations. New Phytol. 2001;149(2):349–55. doi: 10.1046/j.1469-8137.2001.00027.x 33874632

[58] Ruan Y. Flavonoids stimulate spore germination infusarium solanipathogenic on legumes in a manner sensitive to inhibitors of cAMP-dependent protein kinase. Mol Plant-Microbe Interac. 1995;8(6):929. doi: 10.1094/mpmi-8-0929

[59] Straney D, Khan R, Tan R, Bagga S. Host recognition by pathogenic fungi through plant flavonoids. Adv Exp Med Biol. 2002;505:9–22. doi: 10.1007/978-1-4757-5235-9_2 12083470

[60] Westrick NM, Smith DL, Kabbage M. Disarming the host: detoxification of plant defense compounds during fungal necrotrophy. Front Plant Sci. 2021;12:651716. doi: 10.3389/fpls.2021.651716 33995447 PMC8120277

[61] Chen J, Ullah C, Reichelt M, Gershenzon J, Hammerbacher A. Sclerotinia sclerotiorum Circumvents flavonoid defenses by catabolizing flavonol glycosides and aglycones. Plant Physiol. 2019;180(4):1975–87. doi: 10.1104/pp.19.00461 31221733 PMC6670079

[62] Ökmen B, Etalo DW, Joosten MHAJ, Bouwmeester HJ, de Vos RCH, Collemare J, et al. Detoxification of α-tomatine by Cladosporium fulvum is required for full virulence on tomato. New Phytol. 2013;198(4):1203–14. doi: 10.1111/nph.12208 23448507

[63] Patil JR, Mhatre KJ, Yadav K, Yadav LS, Srivastava S, Nikalje GC. Flavonoids in plant-environment interactions and stress responses. Discov Plants. 2024;1(1). doi: 10.1007/s44372-024-00063-6

[64] Lee H, Woo E-R, Lee DG. Apigenin induces cell shrinkage in Candida albicans by membrane perturbation. FEMS Yeast Res. 2018;18(1):10.1093/femsyr/foy003. doi: 10.1093/femsyr/foy003 29346565

