# Supplementary material for: Integrated analysis of genome, metabolome, and transcriptome reveals a bHLH transcription factor potentially regulating the accumulation of flavonoids involved in carrot resistance to Alternaria leaf blight
Source: PLoS One. 2025 Nov 19;20(11):e0336995. doi: 10.1371/journal.pone.0336995 (PMC12629425; doi:10.1371/journal.pone.0336995)
Supplement: S1 File — (PDF) [file pone.0336995.s001.pdf]

Supplementary Information 1

Title: Statistical analyses of apigenin-7-*O*-rutinoside, luteolin-7-*O*-rutinoside, and chrysoeriol-7-*O*-rutinoside accumulations in the leaves of the two carrot genotypes

Statistical analyses supporting the figure 3 of the paper "Integrated analysis of genome, metabolome, and transcriptome reveals a bHLH transcription factor potentially regulating the accumulation of flavonoids involved in carrot resistance to Alternaria leaf blight

Koutouan *et al.*, 2025

Table of contents

Api7R 2
Assumptions verification 2
Log transformation 2
Anova and post-hoc comparisons 3
Lut 7R 4
Assumptions verification 4
Log transformation 4
Anova and post-hoc comparisons 5
Chry 7R 6

```r
library(emmeans)
library(multcomp)
library(car)
```

Api7R

Assumptions verification

```r
mod2=lm(`Api 7R`~Genotype, data=df)
res2=residuals(mod2)
shapiro.test(res2)


    Shapiro-Wilk normality test

data:  res2
W = 0.95867, p-value = 0.7974
```

```r
bartlett.test(res2,g=df$Genotype, data=df)


    Bartlett test of homogeneity of variances

data:  res2 and df$Genotype
Bartlett's K-squared = 7.8434, df = 1, p-value = 0.005101
```

Log transformation

```r
df$logApi7R=log(df$`Api 7R`)
mod3=lm(logApi7R~Genotype, data=df)
res3=residuals(mod3)
shapiro.test(res3)


    Shapiro-Wilk normality test

data:  res3
W = 0.95357, p-value = 0.7471
```

```r
bartlett.test(res3,g=df$Genotype, data=df)


    Bartlett test of homogeneity of variances

data:  res3 and df$Genotype
Bartlett's K-squared = 0.15106, df = 1, p-value = 0.6975
```

Anova and post-hoc comparisons

```
anova(mod3)

Analysis of Variance Table

Response: logApi7R
          Df Sum Sq Mean Sq F value    Pr(>F)
Genotype   1 11.452 11.4517  160.53 1.482e-05 ***
Residuals  6  0.428  0.0713
---
Signif. codes:  0 '***' 0.001 '**' 0.01 '*' 0.05 '.' 0.1 ' ' 1

emmeans_result <- emmeans(mod3, pairwise ~ Genotype , adjust = "tukey",
data=df)
print(emmeans_result)

$emmeans
 Genotype emmean    SE df lower.CL upper.CL
 H1         13.2 0.134  6     12.9     13.5
 I2         15.6 0.134  6     15.3     15.9

Confidence level used: 0.95

$contrasts
 contrast estimate    SE df t.ratio p.value
 H1 - I2     -2.39 0.189  6 -12.670  <.0001

cld_result <- cld(emmeans_result$emmeans, Letters = letters)
cld_result

 Genotype emmean    SE df lower.CL upper.CL .group
 H1         13.2 0.134  6     12.9     13.5  a
 I2         15.6 0.134  6     15.3     15.9   b

Confidence level used: 0.95
significance level used: alpha = 0.05
NOTE: If two or more means share the same grouping symbol,
      then we cannot show them to be different.
      But we also did not show them to be the same.
```

Lut 7R

Assumptions verification

```
mod2=lm(`Lut 7R`~Genotype, data=df)
res2=residuals(mod2)
shapiro.test(res2)


    Shapiro-Wilk normality test

data:  res2
W = 0.90619, p-value = 0.328
```

```
bartlett.test(res2,g=df$Genotype, data=df)


    Bartlett test of homogeneity of variances

data:  res2 and df$Genotype
Bartlett's K-squared = 10.632, df = 1, p-value = 0.001111
```

Log transformation

```
df$logLut7R=log(df$`Lut 7R`)
mod3=lm(logLut7R~Genotype, data=df)
res3=residuals(mod3)
shapiro.test(res3)


    Shapiro-Wilk normality test

data:  res3
W = 0.92274, p-value = 0.4525
```

```
bartlett.test(res3,g=df$Genotype, data=df)


    Bartlett test of homogeneity of variances

data:  res3 and df$Genotype
Bartlett's K-squared = 1.5277, df = 1, p-value = 0.2165
```

Anova and post-hoc comparisons

```
anova(mod3)

Analysis of Variance Table

Response: logLut7R
          Df Sum Sq Mean Sq F value    Pr(>F)
Genotype   1 8.1358  8.1358   100.4 5.727e-05 ***
Residuals  6 0.4862  0.0810
---
Signif. codes:  0 '***' 0.001 '**' 0.01 '*' 0.05 '.' 0.1 ' ' 1

emmeans_result <- emmeans(mod3, pairwise ~ Genotype , adjust = "tukey",
data=df)
print(emmeans_result)

$emmeans
 Genotype emmean    SE df lower.CL upper.CL
 H1         12.9 0.142  6     12.6     13.3
 I2         14.9 0.142  6     14.6     15.3

Confidence level used: 0.95

$contrasts
 contrast estimate    SE df t.ratio p.value
 H1 - I2     -2.02 0.201  6 -10.020  0.0001

cld_result <- cld(emmeans_result$emmeans, Letters = letters)
cld_result

 Genotype emmean    SE df lower.CL upper.CL .group
 H1         12.9 0.142  6     12.6     13.3  a
 I2         14.9 0.142  6     14.6     15.3   b

Confidence level used: 0.95
significance level used: alpha = 0.05
NOTE: If two or more means share the same grouping symbol,
      then we cannot show them to be different.
      But we also did not show them to be the same.
```

Chry 7R

```
mod2=lm(`Chry 7R`~Genotype, data=df)
res2=residuals(mod2)
shapiro.test(res2)


    Shapiro-Wilk normality test

data:  res2
W = 0.81459, p-value = 0.04092
```

```
bartlett.test(res2,g=df$Genotype, data=df)


    Bartlett test of homogeneity of variances

data:  res2 and df$Genotype
Bartlett's K-squared = 14.741, df = 1, p-value = 0.0001234
```

```
df$logChty7R=log(df$`Chry 7R`)
mod3=lm(logChty7R~Genotype, data=df)
res3=residuals(mod3)
shapiro.test(res3)


    Shapiro-Wilk normality test

data:  res3
W = 0.88396, p-value = 0.2054
```

```
bartlett.test(res3,g=df$Genotype, data=df)


    Bartlett test of homogeneity of variances

data:  res3 and df$Genotype
Bartlett's K-squared = 2.85, df = 1, p-value = 0.09138
```

```
emmeans_result <- emmeans(mod3, pairwise ~ Genotype , adjust = "tukey",
data=df)
print(emmeans_result)

$emmeans
 Genotype emmean    SE df lower.CL upper.CL
 H1         12.5 0.132  6     12.2     12.9
 I2         15.1 0.132  6     14.7     15.4
```

```
Confidence level used: 0.95

$contrasts
 contrast estimate    SE df t.ratio p.value
 H1 - I2     -2.53 0.187  6 -13.529  <.0001

cld_result <- cld(emmeans_result$emmeans, Letters = letters)
cld_result

 Genotype emmean    SE df lower.CL upper.CL .group
 H1         12.5 0.132  6     12.2     12.9  a
 I2         15.1 0.132  6     14.7     15.4   b

Confidence level used: 0.95
significance level used: alpha = 0.05
NOTE: If two or more means share the same grouping symbol,
      then we cannot show them to be different.
      But we also did not show them to be the same.
```
