# Supplementary material for: Integrated analysis of genome, metabolome, and transcriptome reveals a bHLH transcription factor potentially regulating the accumulation of flavonoids involved in carrot resistance to Alternaria leaf blight
Source: PLoS One. 2025 Nov 19;20(11):e0336995. doi: 10.1371/journal.pone.0336995 (PMC12629425; doi:10.1371/journal.pone.0336995)
Supplement: S2 File — (PDF) [file pone.0336995.s002.pdf]

Supplementary Information 2

Title: Statistical analyses of expression levels of the *DcbHLH162-like* gene in calli of 11 different H1 transformation events

Statistical analyses supporting the figure 6 of the paper “Integrated analysis of genome, metabolome, and transcriptome reveals a bHLH transcription factor potentially regulating the accumulation of flavonoids involved in carrot resistance to Alternaria leaf blight

Koutouan *et al.*, 2025

Table of contents

Assumptions verification 2
Square Root and Box-Cox transformations 2
Kruskal-Wallis and Conover post-hoc tests 3

```
library(tidyr)
library(emmeans)
library(multcomp)
library(multcompView)
library(PMCMRplus)
library(readxl)
library(car)
library(dplyr)
```

Assumptions verification

```
model=lm(Y~bHLH, data=df)
res=residuals(model)

shapiro.test(res)


    Shapiro-Wilk normality test

data:  res
W = 0.81718, p-value = 1.051e-05

bartlett.test(residuals(model),g=df$bHLH)


    Bartlett test of homogeneity of variances

data:  residuals(model) and df$bHLH
Bartlett's K-squared = 166.75, df = 13, p-value < 2.2e-16
```

Square Root and Box-Cox transformations

```
mod2 <- lm(sqrt(Y)~bHLH, data=df)
res2=residuals(mod2)
bartlett.test(res2, df$bHLH)


    Bartlett test of homogeneity of variances

data:  res2 and df$bHLH
Bartlett's K-squared = 143.21, df = 13, p-value < 2.2e-16

shapiro.test(res2)


    Shapiro-Wilk normality test

data:  res2
W = 0.86841, p-value = 0.0001856

summary(p1<-powerTransform(model))
```

```
bcPower Transformation to Normality
   Est Power Rounded Pwr Wald Lwr Bnd Wald Upr Bnd
Y1    0.1989         0.2       0.1104       0.2874

Likelihood ratio test that transformation parameter is equal to 0
 (log transformation)
                           LRT df       pval
LR test, lambda = (0) 28.43966  1 9.6665e-08

Likelihood ratio test that no transformation is needed
                           LRT df       pval
LR test, lambda = (1) 73.59376  1 < 2.22e-16
```

```
df2=transform(df, Y2=bcPower(Y, coef(p1, round=TRUE)))
mod3 <- lm(Y2~bHLH, data=df2)
res3=residuals(mod3)
bartlett.test(res3, df$bHLH)
```

```
    Bartlett test of homogeneity of variances

data:  res3 and df$bHLH
Bartlett's K-squared = 134.92, df = 13, p-value < 2.2e-16
```

```
shapiro.test(res3)
```

```
    Shapiro-Wilk normality test

data:  res3
W = 0.90437, p-value = 0.00195
```

Kruskal-Wallis and Conover post-hoc tests

```
kw=kruskal.test(Y2~bHLH, data=df2)
kw
```

```
    Kruskal-Wallis rank sum test

data:  Y2 by bHLH
Kruskal-Wallis chi-squared = 40.051, df = 13, p-value = 0.0001357
```

```
posthocConv=kwAllPairsConoverTest(Y~bHLH, data=df,p.adjust.methods="BH")
posthocConv
```

```
           H1 EV   H1 NT   I2 NT   H1 bHLH 11 H1 bHLH 12 H1 bHLH 13 H1 bHLH
10
H1 NT      0.92377 -       -       -          -          -          -
I2 NT      0.12007 0.92377 -       -          -          -          -
H1 bHLH 11 0.00024 0.01547 0.42354 -          -          -          -
```

```
H1 bHLH 12 5.7e-05 0.00397 0.17143 1.00000    -          -          -
H1 bHLH 13 3.6e-06 0.00024 0.01547 0.92377    0.99718    -          -
H1 bHLH 10 4.3e-10 1.4e-08 6.0e-07 0.00038    0.00156    0.02395    -
H1 bHLH 4  1.3e-09 4.6e-08 2.3e-06 0.00156    0.00629    0.08230    1.00000
H1 bHLH 7  1.4e-10 4.1e-09 1.6e-07 9.1e-05    0.00038    0.00629    1.00000
H1 bHLH 5  5.3e-13 9.5e-12 2.1e-10 4.6e-08    1.6e-07    2.3e-06    0.05537
H1 bHLH 6  1.6e-14 3.9e-13 6.8e-12 8.9e-10    2.8e-09    3.1e-08    0.00061
H1 bHLH 9  8.3e-15 2.8e-13 4.9e-12 6.2e-10    1.9e-09    2.0e-08    0.00038
H1 bHLH 1  < 2e-16 < 2e-16 5.6e-14 4.9e-12    1.3e-11    1.0e-10    6.0e-07
H1 bHLH 8  < 2e-16 < 2e-16 < 2e-16 2.8e-13    7.3e-13    4.9e-12    1.4e-08
           H1 bHLH 4 H1 bHLH 7 H1 bHLH 5 H1 bHLH 6 H1 bHLH 9 H1 bHLH 1
H1 NT      -         -         -         -         -         -
I2 NT      -         -         -         -         -         -
H1 bHLH 11 -         -         -         -         -         -
H1 bHLH 12 -         -         -         -         -         -
H1 bHLH 13 -         -         -         -         -         -
H1 bHLH 10 -         -         -         -         -         -
H1 bHLH 4  -         -         -         -         -         -
H1 bHLH 7  0.99718   -         -         -         -         -
H1 bHLH 5  0.01547   0.17143   -         -         -         -
H1 bHLH 6  0.00015   0.00249   0.85498   -         -         -
H1 bHLH 9  9.1e-05   0.00156   0.76192   1.00000   -         -
H1 bHLH 1  1.6e-07   2.3e-06   0.00629   0.32312   0.42354   -
H1 bHLH 8  4.1e-09   4.6e-08   9.1e-05   0.00990   0.01547   0.92377
```

```
conover_result <- kwAllPairsConoverTest(Y ~ bHLH, data = df, p.adjust.method
= "BH")
```

```
Warning in kwAllPairsConoverTest.default(c(1, 1.38991821984234,
115.741457355142, : Ties are present. Quantiles were corrected for ties.
```

```
conover_result
```

```


    Pairwise comparisons using Conover's all-pairs test

data: Y by bHLH

           H1 EV   H1 NT   I2 NT   H1 bHLH 11 H1 bHLH 12 H1 bHLH 13 H1 bHLH
10
H1 NT      0.12310 -       -       -          -          -          -
I2 NT      0.00366 0.12310 -       -          -          -          -
H1 bHLH 11 5.6e-06 0.00036 0.01992 -          -          -          -
H1 bHLH 12 1.5e-06 9.3e-05 0.00561 0.59850    -          -          -
H1 bHLH 13 9.0e-08 5.6e-06 0.00036 0.12310    0.30059    -          -
H1 bHLH 10 2.0e-11 4.7e-10 1.6e-08 8.7e-06    3.6e-05    0.00057    -
H1 bHLH 4  5.5e-11 1.4e-09 5.8e-08 3.6e-05    0.00014    0.00233    0.59850
H1 bHLH 7  7.4e-12 1.5e-10 4.6e-09 2.3e-06    8.7e-06    0.00014    0.59850
H1 bHLH 5  4.9e-14 5.6e-13 1.0e-11 1.4e-09    4.6e-09    5.8e-08    0.00148
H1 bHLH 6  4.8e-15 4.0e-14 4.3e-13 3.9e-11    1.1e-10    9.9e-10    1.4e-05
H1 bHLH 9  4.2e-15 3.3e-14 3.3e-13 2.8e-11    7.7e-11    6.8e-10    8.7e-06
```

```
H1 bHLH 1  2.3e-16 1.3e-15 9.6e-15 3.3e-13    7.4e-13    5.5e-12    1.6e-08
H1 bHLH 8  < 2e-16 2.3e-16 1.3e-15 3.3e-14    6.2e-14    3.3e-13    4.7e-10
           H1 bHLH 4 H1 bHLH 7 H1 bHLH 5 H1 bHLH 6 H1 bHLH 9 H1 bHLH 1
H1 NT      -         -         -         -         -         -
I2 NT      -         -         -         -         -         -
H1 bHLH 11 -         -         -         -         -         -
H1 bHLH 12 -         -         -         -         -         -
H1 bHLH 13 -         -         -         -         -         -
H1 bHLH 10 -         -         -         -         -         -
H1 bHLH 4  -         -         -         -         -         -
H1 bHLH 7  0.30059   -         -         -         -         -
H1 bHLH 5  0.00036   0.00561   -         -         -         -
H1 bHLH 6  3.6e-06   5.7e-05   0.09149   -         -         -
H1 bHLH 9  2.3e-06   3.6e-05   0.06442   0.85786   -         -
H1 bHLH 1  4.6e-09   5.8e-08   0.00014   0.01338   0.01992   -
H1 bHLH 8  1.5e-10   1.4e-09   2.3e-06   0.00023   0.00036   0.12310


P value adjustment method: BH
```

```
pvals <- conover_result$p.value
pvals_df <- as.data.frame(as.table(pvals)) %>%
  filter(!is.na(Freq)) %>%
  rename(Group1 = Var1, Group2 = Var2, p_value = Freq) %>%
  arrange(p_value)

group_names <- unique(c(pvals_df$Group1, pvals_df$Group2))
pval_matrix_conover <- matrix(1, nrow = length(group_names), ncol =
length(group_names),
                      dimnames = list(group_names, group_names))
diag(pval_matrix_conover) <- 1

 for (i in 1:nrow(pvals_df)) {
  g1 <- as.character(pvals_df$Group1[i])
  g2 <- as.character(pvals_df$Group2[i])

  pval_matrix_conover[g1, g2] <- pvals_df$p_value[i]
  pval_matrix_conover[g2, g1] <- pvals_df$p_value[i]
}
pval_matrix_conover
```

```
              H1 bHLH 8    H1 bHLH 1    H1 bHLH 9    H1 bHLH 6    H1 bHLH 5
H1 bHLH 8  1.000000e+00 1.231019e-01 3.577522e-04 2.326474e-04 2.252897e-06
H1 bHLH 1  1.231019e-01 1.000000e+00 1.991722e-02 1.337835e-02 1.445019e-04
H1 bHLH 9  3.577522e-04 1.991722e-02 1.000000e+00 8.578624e-01 6.442168e-02
H1 bHLH 6  2.326474e-04 1.337835e-02 8.578624e-01 1.000000e+00 9.148691e-02
H1 bHLH 5  2.252897e-06 1.445019e-04 6.442168e-02 9.148691e-02 1.000000e+00
H1 bHLH 7  1.382591e-09 5.790341e-08 3.558919e-05 5.738284e-05 5.612759e-03
H1 bHLH 10 4.658858e-10 1.610604e-08 8.691021e-06 1.396215e-05 1.475191e-03
H1 bHLH 4  1.504394e-10 4.561285e-09 2.252897e-06 3.589019e-06 3.577522e-04
```

```
H1 bHLH 13 3.274272e-13 5.458048e-12 6.785034e-10 9.932816e-10 5.790341e-08
H1 bHLH 12 6.156940e-14 7.359477e-13 7.693675e-11 1.091304e-10 4.561285e-09
H1 bHLH 11 3.255608e-14 3.274272e-13 2.785558e-11 3.886757e-11 1.382591e-09
I2 NT      1.335895e-15 9.598659e-15 3.274272e-13 4.260837e-13 1.012888e-11
H1 NT      2.320212e-16 1.335895e-15 3.255608e-14 3.983248e-14 5.581950e-13
H1 EV      8.494365e-17 2.320212e-16 4.220836e-15 4.756441e-15 4.927966e-14
              H1 bHLH 7   H1 bHLH 10    H1 bHLH 4   H1 bHLH 13   H1 bHLH 12
H1 bHLH 8  1.382591e-09 4.658858e-10 1.504394e-10 3.274272e-13 6.156940e-14
H1 bHLH 1  5.790341e-08 1.610604e-08 4.561285e-09 5.458048e-12 7.359477e-13
H1 bHLH 9  3.558919e-05 8.691021e-06 2.252897e-06 6.785034e-10 7.693675e-11
H1 bHLH 6  5.738284e-05 1.396215e-05 3.589019e-06 9.932816e-10 1.091304e-10
H1 bHLH 5  5.612759e-03 1.475191e-03 3.577522e-04 5.790341e-08 4.561285e-09
H1 bHLH 7  1.000000e+00 5.985044e-01 3.005950e-01 1.445019e-04 8.691021e-06
H1 bHLH 10 5.985044e-01 1.000000e+00 5.985044e-01 5.734373e-04 3.558919e-05
H1 bHLH 4  3.005950e-01 5.985044e-01 1.000000e+00 2.330103e-03 1.445019e-04
H1 bHLH 13 1.445019e-04 5.734373e-04 2.330103e-03 1.000000e+00 3.005950e-01
H1 bHLH 12 8.691021e-06 3.558919e-05 1.445019e-04 3.005950e-01 1.000000e+00
H1 bHLH 11 2.252897e-06 8.691021e-06 3.558919e-05 1.231019e-01 5.985044e-01
I2 NT      4.561285e-09 1.610604e-08 5.790341e-08 3.577522e-04 5.612759e-03
H1 NT      1.504394e-10 4.658858e-10 1.382591e-09 5.625587e-06 9.252330e-05
H1 EV      7.413163e-12 2.007634e-11 5.453458e-11 8.953009e-08 1.479012e-06
             H1 bHLH 11        I2 NT        H1 NT        H1 EV
H1 bHLH 8  3.255608e-14 1.335895e-15 2.320212e-16 8.494365e-17
H1 bHLH 1  3.274272e-13 9.598659e-15 1.335895e-15 2.320212e-16
H1 bHLH 9  2.785558e-11 3.274272e-13 3.255608e-14 4.220836e-15
H1 bHLH 6  3.886757e-11 4.260837e-13 3.983248e-14 4.756441e-15
H1 bHLH 5  1.382591e-09 1.012888e-11 5.581950e-13 4.927966e-14
H1 bHLH 7  2.252897e-06 4.561285e-09 1.504394e-10 7.413163e-12
H1 bHLH 10 8.691021e-06 1.610604e-08 4.658858e-10 2.007634e-11
H1 bHLH 4  3.558919e-05 5.790341e-08 1.382591e-09 5.453458e-11
H1 bHLH 13 1.231019e-01 3.577522e-04 5.625587e-06 8.953009e-08
H1 bHLH 12 5.985044e-01 5.612759e-03 9.252330e-05 1.479012e-06
H1 bHLH 11 1.000000e+00 1.991722e-02 3.577522e-04 5.625587e-06
I2 NT      1.991722e-02 1.000000e+00 1.231019e-01 3.655111e-03
H1 NT      3.577522e-04 1.231019e-01 1.000000e+00 1.231019e-01
H1 EV      5.625587e-06 3.655111e-03 1.231019e-01 1.000000e+00
```

```
conover_letters=multcompLetters(pval_matrix_conover)
conover_letters
```

```
 H1 bHLH 8  H1 bHLH 1  H1 bHLH 9  H1 bHLH 6  H1 bHLH 5  H1 bHLH 7 H1 bHLH 10 
       "a"        "a"        "b"        "b"        "b"        "c"        "c" 
 H1 bHLH 4 H1 bHLH 13 H1 bHLH 12 H1 bHLH 11      I2 NT      H1 NT      H1 EV 
       "c"        "d"        "d"        "d"        "e"       "ef"        "f" 
```
