# Supplementary material for: Integrated analysis of genome, metabolome, and transcriptome reveals a bHLH transcription factor potentially regulating the accumulation of flavonoids involved in carrot resistance to Alternaria leaf blight
Source: PLoS One. 2025 Nov 19;20(11):e0336995. doi: 10.1371/journal.pone.0336995 (PMC12629425; doi:10.1371/journal.pone.0336995)
Supplement: S3 File — (PDF) [file pone.0336995.s003.pdf]

Supplementary Information 3

Title: Statistical analyses of accumulation levels of luteolin-7-*O*-rutinoside, and chrysoeriol-7-*O*-rutinoside in the calli of the two native or transformed carrot genotypes H1 and I2

Statistical analyses supporting the figure 7 of the paper “Integrated analysis of genome, metabolome, and transcriptome reveals a bHLH transcription factor potentially regulating the accumulation of flavonoids involved in carrot resistance to Alternaria leaf blight

Koutouan *et al.*, 2025

Table of contents

Lut 7R....2
Assumptions verification....2
Kruskal-Wallis and Conover post-hoc tests....3
Chry 7R....4
Assumptions verification....4
Kruskal-Wallis and Conover post-hoc tests....5

```
library(car)
library(multcomp)
library(PMCMRplus)
library(multcompView)
library(dplyr)
```

Lut 7R

Assumptions verification

```
mod1=lm(Lut7R~genotype, data=df)
res=residuals(mod1)
```

Box-Cox transformation.

```
summary(p1 <- powerTransform(mod1))

bcPower Transformation to Normality
   Est Power Rounded Pwr Wald Lwr Bnd Wald Upr Bnd
Y1    0.1711        0.17        0.027       0.3153

Likelihood ratio test that transformation parameter is equal to 0
 (log transformation)
                           LRT df     pval
LR test, lambda = (0) 5.198141  1 0.022611

Likelihood ratio test that no transformation is needed
                           LRT df       pval
LR test, lambda = (1) 69.72871  1 < 2.22e-16

df_bc <- transform(df, Lut7R_bc=bcPower(Lut7R,coef(p1)))
head(df_bc)

  genotype    Lut7R    Chry7R Lut7R_bc
1    H1 NT 1000.000  1000.000 13.21338
2    H1 NT 1000.000  1000.000 13.21338
3    H1 NT 1878.729  1000.000 15.38486
4    H1 NT 1000.000  1306.917 13.21338
5    H1 NT 2584.241 13565.422 16.57526
6    I2 NT 1000.000  1000.000 13.21338

mod2=lm(Lut7R_bc~genotype, data=df_bc)
res=residuals(mod2)
shapiro.test(res)


    Shapiro-Wilk normality test

data:  res
W = 0.96479, p-value = 0.6172
```

```
bartlett.test(res, g=df$genotype, data=df)


    Bartlett test of homogeneity of variances

data:  res and df$genotype
Bartlett's K-squared = 12.68, df = 2, p-value = 0.001764
```

Kruskal-Wallis and Conover post-hoc tests

```
mkw=kruskal.test(Lut7R~genotype, data=df)
mkw


    Kruskal-Wallis rank sum test

data:  Lut7R by genotype
Kruskal-Wallis chi-squared = 15.945, df = 2, p-value = 0.0003448

res=kwAllPairsConoverTest(Lut7R~genotype, data=df)

Warning in kwAllPairsConoverTest.default(c(1000, 1000, 1878.72920074585, :
Ties
are present. Quantiles were corrected for ties.

res


    Pairwise comparisons using Conover's all-pairs test

data: Lut7R by genotype

      H1 bHLH H1 NT
H1 NT 1.1e-06 -
I2 NT 9.1e-05 0.16


P value adjustment method: single-step

pvals <- res$p.value
pvals_df <- as.data.frame(as.table(pvals)) %>%
  filter(!is.na(Freq)) %>%
  rename(Group1 = Var1, Group2 = Var2, p_value = Freq) %>%
  arrange(p_value)

group_names <- unique(c(pvals_df$Group1, pvals_df$Group2))
pval_matrix <- matrix(1, nrow = length(group_names), ncol =
length(group_names),
                      dimnames = list(group_names, group_names))
diag(pval_matrix) <- 1

 for (i in 1:nrow(pvals_df)) {
```

```
  g1 <- as.character(pvals_df$Group1[i])
  g2 <- as.character(pvals_df$Group2[i])

  pval_matrix[g1, g2] <- pvals_df$p_value[i]
  pval_matrix[g2, g1] <- pvals_df$p_value[i]
}

pval_matrix

               H1 NT        I2 NT      H1 bHLH
H1 NT   1.000000e+00 0.1565959241 1.071939e-06
I2 NT   1.565959e-01 1.0000000000 9.134230e-05
H1 bHLH 1.071939e-06 0.0000913423 1.000000e+00

multcompLetters(pval_matrix)

  H1 NT   I2 NT H1 bHLH 
    "a"     "a"     "b" 
```

Chry 7R

Assumptions verification

```
mod1=lm(Chry7R~genotype, data=df)
res=residuals(mod1)
shapiro.test(res)


    Shapiro-Wilk normality test

data:  res
W = 0.8395, p-value = 0.002816

bartlett.test(res, g=df$genotype, data=df)


    Bartlett test of homogeneity of variances

data:  res and df$genotype
Bartlett's K-squared = 41.915, df = 2, p-value = 7.91e-10
```

Box-Cox transformation.

```
summary(p1 <- powerTransform(mod1))

bcPower Transformation to Normality 
   Est Power Rounded Pwr Wald Lwr Bnd Wald Upr Bnd
Y1    0.1287           0      -0.0603       0.3178

Likelihood ratio test that transformation parameter is equal to 0
```

```
 (log transformation)
                           LRT df    pval
LR test, lambda = (0) 1.804981  1 0.17911

Likelihood ratio test that no transformation is needed
                           LRT df       pval
LR test, lambda = (1) 51.54962  1 6.9811e-13
```

```
df$LogChry7R=log(df$Chry7R)
mod2=lm(LogChry7R~genotype, data=df)
res=residuals(mod2)
shapiro.test(res)
```

```

    Shapiro-Wilk normality test

data:  res
W = 0.80733, p-value = 0.0008509
```

```
bartlett.test(res, g=df$genotype, data=df)
```

```

    Bartlett test of homogeneity of variances

data:  res and df$genotype
Bartlett's K-squared = 17.001, df = 2, p-value = 0.0002033
```

Kruskal-Wallis and Conover post-hoc tests

```
mkw=kruskal.test(Chry7R~genotype, data=df)
mkw
```

```

    Kruskal-Wallis rank sum test

data:  Chry7R by genotype
Kruskal-Wallis chi-squared = 9.1762, df = 2, p-value = 0.01017
```

```
res=kwAllPairsConoverTest(Chry7R~genotype, data=df)
```

```
Warning in kwAllPairsConoverTest.default(c(1000, 1000, 1000,
1306.91665293777,
: Ties are present. Quantiles were corrected for ties.
```

```
pvals <- res$p.value
pvals_df <- as.data.frame(as.table(pvals)) %>%
  filter(!is.na(Freq)) %>%
  rename(Group1 = Var1, Group2 = Var2, p_value = Freq) %>%
  arrange(p_value)

group_names <- unique(c(pvals_df$Group1, pvals_df$Group2))
```

```r
pval_matrix <- matrix(1, nrow = length(group_names), ncol =
length(group_names),
                      dimnames = list(group_names, group_names))
diag(pval_matrix) <- 1

 for (i in 1:nrow(pvals_df)) {
  g1 <- as.character(pvals_df$Group1[i])
  g2 <- as.character(pvals_df$Group2[i])

  pval_matrix[g1, g2] <- pvals_df$p_value[i]
  pval_matrix[g2, g1] <- pvals_df$p_value[i]
}

pval_matrix

              H1 NT       I2 NT      H1 bHLH
H1 NT   1.000000000 0.01064133 0.005167838
I2 NT   0.010641331 1.00000000 0.969947639
H1 bHLH 0.005167838 0.96994764 1.000000000

multcompLetters(pval_matrix)

  H1 NT   I2 NT H1 bHLH
    "a"     "b"     "b"
```
