# Supplementary material for: Integrated analysis of genome, metabolome, and transcriptome reveals a bHLH transcription factor potentially regulating the accumulation of flavonoids involved in carrot resistance to Alternaria leaf blight
Source: PLoS One. 2025 Nov 19;20(11):e0336995. doi: 10.1371/journal.pone.0336995 (PMC12629425; doi:10.1371/journal.pone.0336995)
Supplement: S4 File — (PDF) [file pone.0336995.s004.pdf]

Supplementary Information 4

Title: Statistical analyses of germination and disruption percentages calculated from 3200 microscope observations of conidia exposed to callus extracts or control solution

Statistical analyses supporting the figure 8A of the paper “Integrated analysis of genome, metabolome, and transcriptome reveals a bHLH transcription factor potentially regulating the accumulation of flavonoids involved in carrot resistance to Alternaria leaf blight

Koutouan *et al.*, 2025

Table of contents

Biological assay data analysis 2

Response variable: germination percentage 2

Assumptions verification 2

Anova and post-hoc comparisons 2

Response variable: disruption percentage 3

Assumptions verification 3

Anova and post-hoc comparisons 3

Biological assay data analysis

Response variable: germination percentage

```
model=lm(germination~Facteur, data=Exp5)
residus=residuals(model)
```

Assumptions verification

```
shapiro.test(residus)


    Shapiro-Wilk normality test

data:  residus
W = 0.9392, p-value = 0.3393

bartlett.test(hyphes ~ Facteur, data = Exp5)


    Bartlett test of homogeneity of variances

data:  hyphes by Facteur
Bartlett's K-squared = 7.1523, df = 3, p-value = 0.0672
```

Anova and post-hoc comparisons

```
anova(model)

Analysis of Variance Table

Response: germination
          Df Sum Sq Mean Sq F value    Pr(>F)    
Facteur    3 7229.7 2409.90  119.38 3.358e-09 ***
Residuals 12  242.2   20.19                      
---
Signif. codes:  0 '***' 0.001 '**' 0.01 '*' 0.05 '.' 0.1 ' ' 1

tukey_result =TukeyHSD(aov(germination ~  Facteur, data=Exp5))
tukey_result

  Tukey multiple comparisons of means
    95% family-wise confidence level

Fit: aov(formula = germination ~ Facteur, data = Exp5)

$Facteur
              diff       lwr         upr     p adj
H1_bHLH-DMSO 54.00  44.567604 63.43239557 0.0000000
H1NT-DMSO    44.50  35.067604 53.93239557 0.0000000
I2NT-DMSO    46.75  37.317604 56.18239557 0.0000000
```

```
H1NT-H1_bHLH -9.50 -18.932396 -0.06760443 0.0481954
I2NT-H1_bHLH -7.25 -16.682396  2.18239557 0.1569480
I2NT-H1NT     2.25  -7.182396 11.68239557 0.8919276
```

```
testmultp <- emmeans(model, "Facteur")
cld(testmultp, Letter="abcdefg")
```

```
 Facteur emmean   SE df lower.CL upper.CL .group
 DMSO      36.2 2.25 12     31.4     41.1  a    
 H1NT      80.8 2.25 12     75.9     85.6   b   
 I2NT      83.0 2.25 12     78.1     87.9   bc  
 H1_bHLH   90.2 2.25 12     85.4     95.1    c  

Confidence level used: 0.95 
P value adjustment: tukey method for comparing a family of 4 estimates 
significance level used: alpha = 0.05 
NOTE: If two or more means share the same grouping symbol,
      then we cannot show them to be different.
      But we also did not show them to be the same.
```

Response variable: disruption percentage

```
model=lm(disruption~Facteur, data=Exp5)
residus=residuals(model)
```

Assumptions verification

```
shapiro.test(residus)
```

```

    Shapiro-Wilk normality test

data:  residus
W = 0.97228, p-value = 0.874
```

```
bartlett.test(disruption ~ Facteur, data = Exp5)
```

```

    Bartlett test of homogeneity of variances

data:  disruption by Facteur
Bartlett's K-squared = 1.4763, df = 3, p-value = 0.6878
```

Anova and post-hoc comparisons

```
anova(model)
```

```
Analysis of Variance Table

Response: disruption
          Df  Sum Sq Mean Sq F value    Pr(>F)    
Facteur    3 13833.7  4611.2  247.86 4.674e-11 ***
```

```
Residuals 12   223.2    18.6
---
Signif. codes:  0 '***' 0.001 '**' 0.01 '*' 0.05 '.' 0.1 ' ' 1

tukey_result =TukeyHSD(aov(disruption ~  Facteur, data=Exp5))
tukey_result

  Tukey multiple comparisons of means
    95% family-wise confidence level

Fit: aov(formula = disruption ~ Facteur, data = Exp5)

$Facteur
               diff        lwr        upr     p adj
H1_bHLH-DMSO  74.50  65.445054 83.5549456 0.0000000
H1NT-DMSO     60.50  51.445054 69.5549456 0.0000000
I2NT-DMSO     65.75  56.695054 74.8049456 0.0000000
H1NT-H1_bHLH -14.00 -23.054946 -4.9450544 0.0030048
I2NT-H1_bHLH  -8.75 -17.804946  0.3049456 0.0593848
I2NT-H1NT      5.25  -3.804946 14.3049456 0.3552293

testmultp <- emmeans(model, "Facteur")
cld(testmultp, Letter="abcdefg")

 Facteur emmean   SE df lower.CL upper.CL .group
 DMSO      9.75 2.16 12     5.05     14.4  a    
 H1NT     70.25 2.16 12    65.55     74.9   b   
 I2NT     75.50 2.16 12    70.80     80.2   bc  
 H1_bHLH  84.25 2.16 12    79.55     88.9    c  

Confidence level used: 0.95
P value adjustment: tukey method for comparing a family of 4 estimates
significance level used: alpha = 0.05
NOTE: If two or more means share the same grouping symbol,
      then we cannot show them to be different.
      But we also did not show them to be the same.
```
